# Conditional Inactivation of the DNA Damage Response Gene *Hus1* in Mouse Testis Reveals Separable Roles for Components of the RAD9-RAD1-HUS1 Complex in Meiotic Chromosome Maintenance

**DOI:** 10.1371/journal.pgen.1003320

**Published:** 2013-02-28

**Authors:** Amy M. Lyndaker, Pei Xin Lim, Joanna M. Mleczko, Catherine E. Diggins, J. Kim Holloway, Rebecca J. Holmes, Rui Kan, Donald H. Schlafer, Raimundo Freire, Paula E. Cohen, Robert S. Weiss

**Affiliations:** 1 Department of Biomedical Sciences, Cornell University, Ithaca, New York, United States of America; 2 Unidad de Investigación, Hospital Universitario de Canarias, Instituto de Tecnologias Biomedicas, Tenerife, Spain; The University of North Carolina at Chapel Hill, United States of America

## Abstract

The RAD9-RAD1-HUS1 (9-1-1) complex is a heterotrimeric PCNA-like clamp that responds to DNA damage in somatic cells by promoting DNA repair as well as ATR-dependent DNA damage checkpoint signaling. In yeast, worms, and flies, the 9-1-1 complex is also required for meiotic checkpoint function and efficient completion of meiotic recombination; however, since *Rad9*, *Rad1*, and *Hus1* are essential genes in mammals, little is known about their functions in mammalian germ cells. In this study, we assessed the meiotic functions of 9-1-1 by analyzing mice with germ cell-specific deletion of *Hus1* as well as by examining the localization of RAD9 and RAD1 on meiotic chromosomes during prophase I. *Hus1* loss in testicular germ cells resulted in meiotic defects, germ cell depletion, and severely compromised fertility. *Hus1-*deficient primary spermatocytes exhibited persistent autosomal γH2AX and RAD51 staining indicative of unrepaired meiotic DSBs, synapsis defects, an extended XY body domain often encompassing partial or whole autosomes, and an increase in structural chromosome abnormalities such as end-to-end X chromosome-autosome fusions and ruptures in the synaptonemal complex. Most of these aberrations persisted in diplotene-stage spermatocytes. Consistent with a role for the 9-1-1 complex in meiotic DSB repair, RAD9 localized to punctate, RAD51-containing foci on meiotic chromosomes in a *Hus1*-dependent manner. Interestingly, RAD1 had a broader distribution that only partially overlapped with RAD9, and localization of both RAD1 and the ATR activator TOPBP1 to the XY body and to unsynapsed autosomes was intact in *Hus1* conditional knockouts. We conclude that mammalian HUS1 acts as a component of the canonical 9-1-1 complex during meiotic prophase I to promote DSB repair and further propose that RAD1 and TOPBP1 respond to unsynapsed chromatin through an alternative mechanism that does not require RAD9 or HUS1.

## Author Summary

Meiosis is a specialized cell division process in which germ cells undergo two cell divisions to produce haploid progeny. Two processes, genetic recombination and chromosome pairing/synapsis, are critical for successful meiosis and the production of gametes with high chromosomal integrity. The RAD9-RAD1-HUS1 (9-1-1) complex has been proposed to play critical roles in recombination as well as in the checkpoint-dependent monitoring of chromosomal synapsis by facilitating activation of the ATR checkpoint kinase. Our data indicate that HUS1 is required for normal germ cell development and fertility, for efficient completion of a subset of meiotic DNA recombination events, and for proper exclusion of the non-sex chromosomes from a specialized, repressive chromatin domain containing the X and Y chromosomes. However, HUS1 is not required for the meiotic functions of ATR in responding to chromosome synapsis defects. Furthermore, RAD1 localizes to sites along asynapsed chromosomes that lack detectable RAD9, and does so in the absence of *Hus1*, implicating RAD1 in a novel response to unsynapsed chromatin that is independent of the canonical 9-1-1 complex. Since mice lacking *Hus1* in germ cells exhibit chromosomal abnormalities and severely reduced fertility, this work has broad implications for the maintenance of genome stability in the germline and for human reproductive health.

## Introduction

The requirement for effective genome maintenance is particularly notable in germ cells, which must transmit high quality DNA to future generations. Therefore, germ cells must employ DNA damage response mechanisms that are at least as stringent as those present in somatic cells. Meiosis, which includes the intentional generation and subsequent repair of DNA double-strand breaks (DSBs), involves a variety of DNA repair mechanisms as well as cell cycle checkpoints that monitor chromosomal integrity; however, much remains unknown about how these mechanisms operate in mammalian cells. In this study, we investigated how an essential DNA repair and DNA damage checkpoint complex, the RAD9-RAD1-HUS1 (9-1-1) complex, functions to maintain genome integrity in the germline.

The 9-1-1 complex is a heterotrimeric ring that shares extensive structural similarity with PCNA, the sliding clamp that functions in DNA replication and repair [1]. In mammalian somatic cells, the best characterized role of 9-1-1 is in activation of ATR pathway DNA damage checkpoint signaling following replication-associated DNA damage [2]. In response to stalled replication forks or lesions involving single-stranded DNA, the 9-1-1 complex is loaded onto 5′ recessed ends by the RAD17-RFC clamp loader complex. The ATR kinase is independently loaded onto RPA-coated single-stranded DNA through the interaction of ATR-interacting protein (ATRIP) with both ATR and RPA [3]. The phosphorylated RAD9 C-terminus physically recruits the ATR activator TOPBP1, which then stimulates the kinase activity of ATR [reviewed in 4]–[6]. Once active, ATR phosphorylates downstream effectors such as CHK1 that promote cell cycle arrest, DNA repair, or apoptosis.

In addition to its known checkpoint signaling function, the 9-1-1 clamp physically interacts with a variety of DNA repair proteins, indicating that, like PCNA, 9-1-1 may also function as a scaffold for recruiting DNA repair proteins to damage sites. Indeed, 9-1-1 has been shown to interact with RAD51 in human cells [7], and 9-1-1 complexes from yeast, mouse, and human physically interact with, and in some cases, stimulate the activity of, translesion polymerases [8] as well as base excision repair factors, including DNA Polymerase β [9], [10], FEN1 [11], [12], APE1 [10], DNA ligase I [13], and the NEIL1, TDG, OGG1, and MutY glycosylases [14]–[18]. Other 9-1-1 physical interactors are as varied as HDAC1 histone deacetylase [19], WRN helicase [20], mismatch repair factors MSH2, MSH3, MSH6, and MLH1 [21], [22], and the ATR co-activator RHINO [23]. That 9-1-1 interacts with and stimulates numerous DNA repair factors points to a direct role of 9-1-1 in facilitating DNA repair apart from its ATR-dependent checkpoint signaling activities, though *in vivo* evidence for this proposed function for the mammalian 9-1-1 complex remains limited.

During meiosis, homologous chromosomes pair, synapse, and undergo recombination. The ATR signaling pathway has been proposed to monitor both meiotic chromosomal synapsis and recombination as part of the pachytene DNA damage checkpoint that is believed to prevent cells with unrepaired breaks or synapsis defects from progressing beyond meiotic prophase I. ATR and its activator TOPBP1 localize to unsynapsed autosome cores in zygotene spermatocytes and to the sex body domain during pachytene [24]–[26], where the mostly unsynapsed X and Y chromosomes in males are packaged into a unique chromatin territory [27], [28]. Phosphorylation of histone H2AX to γH2AX, which marks the entire sex body domain, is thought to be ATR-dependent, whereas H2AX phosphorylation at DSB sites on autosomes during leptotene and early zygotene stages appears to depend primarily on another checkpoint kinase, ATM [26], [29]. In the sex body, ATR promotes transcriptional silencing of the X and Y chromosomes in a process termed meiotic sex chromosome inactivation (MSCI) [30], which is required for male fertility [31]. ATR and TOPBP1 also promote meiotic silencing of unsynapsed chromatin (MSUC), a process fundamentally similar to MSCI that functions at sites of autosomal asynapsis during pachytene [24], [25], [30], [32]. MSUC and MSCI require BRCA1 loading at asynapsed chromatin, and both BRCA1 and the HORMA domain-containing protein HORMAD2 must be present at asynapsed axes in order for ATR to be recruited [26], [33]. While TOPBP1 localizes to the sex body and sites of failed autosomal synapsis and is a known ATR activator, it is unclear whether it is strictly required for MSCI and MSUC, and also whether its recruitment is dependent upon the 9-1-1 complex, as during somatic responses to replication stress. The canonical model for mammalian ATR activation predicts a requirement for 9-1-1 in meiotic ATR activation, perhaps after HORMAD2 or BRCA1 loading, although this has remained untested until this study.

Studies in several organisms indicate that the 9-1-1 complex plays critical roles during meiosis, not only for activation of the pachytene meiotic checkpoint in response to DNA damage or synapsis defects [34]–[36], but also in DSB repair. Yeast *rad17* (m*Rad1*) mutants exhibit persistent RAD51 foci [37], in addition to increased rates of ectopic recombination and altered crossover frequencies during meiosis [38], and DDC1 (mRAD9) colocalizes with RAD51 [35]. In *Drosophila*, meiotic DSBs are not repaired efficiently in the absence of *Hus1* [39]. Additionally, *Spo11-*deficient mouse mutants, which lack meiotic DSBs, have reduced *Hus1* expression, suggesting that 9-1-1 functions depend upon DSB formation [40]. The same is true for expression of *Mre11* and *Brca2*, which are known to be involved in DSB repair, further supporting the idea that 9-1-1 may function at meiotic DSBs.

The existing evidence from lower eukaryotes, together with reported physical interactions with mammalian DNA repair and meiotic proteins, suggests a possible critical role for the 9-1-1 complex during mammalian meiosis, either through activation of ATR-dependent checkpoint functions or direct roles in DNA repair at damage sites. Although mouse RAD1 has been determined to localize to both synapsed and unsynapsed meiotic chromosomes [41], little is known about the requirements for 9-1-1 complex components in mammalian germ cells because mutation of *Rad9*, *Rad1*, or *Hus1* results in embryonic lethality [42]–[45]. We addressed this by producing mice with germ cell-specific *Hus1* deletion. Two conditional knockout models were generated, utilizing *Stra8-Cre* expressed in spermatogonia and *Spo11-Cre* expressed in spermatocytes, and in both cases, *Hus1* inactivation in testicular germ cells resulted in persistent meiotic DNA damage, chromosomal defects, and germ cell depletion. Meiotic silencing, on the other hand, appeared unaffected, indicating that HUS1 is dispensable for at least some meiotic ATR functions, including meiotic sex chromosome inactivation. Intriguingly, *Hus1* inactivation resulted in complete loss of RAD9 but not RAD1 foci from meiotic chromosomes, suggesting that RAD1 has additional meiotic functions, likely related to the monitoring of unsynapsed chromatin based on its localization pattern. Together, these data indicate that efficient DSB repair, proper maintenance of the sex body domain, and preservation of chromosome integrity during meiosis require HUS1, and additionally highlight novel roles for 9-1-1 components outside of the conventional heterotrimeric complex.

## Results

### Generation of germ cell-specific *Hus1* conditional knockout mice

**Figure 1:**
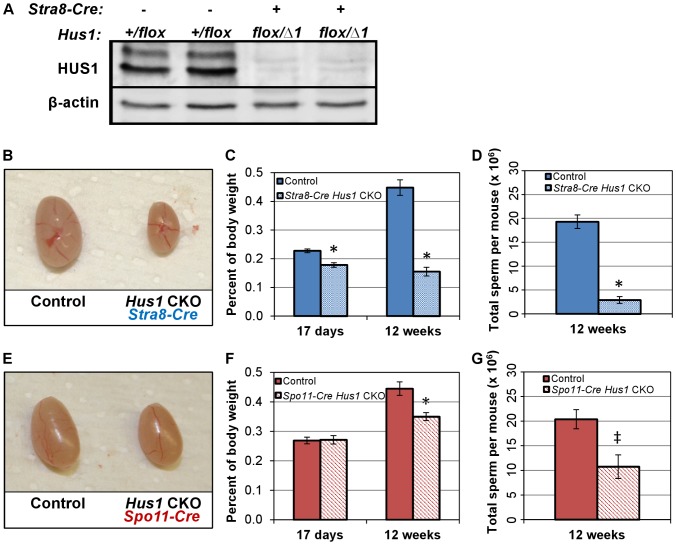
Conditional *Hus1* inactivation in the mouse testis results in reduced testis size and sperm count. A. Western blot analysis of HUS1 protein in adult (12-week old) control and *Hus1* CKO testes. B, E. Photographs of testes from 12-week old *Hus1* CKO and control mice from *Stra8-Cre* and *Spo11-Cre* crosses, respectively. In these representative images, the *Stra8-Cre* control is *Cre*-positive *Hus1^+/flox^*, whereas the *Spo11-Cre* control is *Cre*-negative *Hus1^flox/Δ1^*. C, F. Testis weights of 17-day and 12-week old adult *Hus1* CKO and control mice from both *Stra8-Cre* and *Spo11-Cre* crosses, shown as the mean testis weight relative to body weight ± SEM. D, G. Epididymal sperm counts from *Stra8-Cre* and *Spo11-Cre Hus1* CKO 12-week old males, shown as the mean ± SEM. Statistically significant differences between *Hus1* CKO and the respective control as determined by Student's *t*-test are indicated (* at p<0.001; ^‡^ at p<0.01).

Several lines of evidence from non-vertebrate organisms suggest that the mammalian 9-1-1 complex, the components of which are highly expressed in mouse and human testis, is likely to be critical for normal germ cell development and completion of meiosis. In order to assess the meiotic functions of the 9-1-1 complex, we generated mice in which the *Hus1* gene was conditionally inactivated specifically in testicular germ cells. We combined a conditional floxed *Hus1* allele (Figure S1A; [46]) with two *Cre*-expressing lines, *Stra8-Cre* and *Spo11-Cre*. *Stra8-Cre* mice begin to express the CRE recombinase in spermatogonia [47], which undergo several rounds of mitotic division prior to meiosis, whereas CRE expression in *Spo11-Cre* mice begins in spermatocytes that have initiated meiosis (Figures S1 and S2; Text S1). We focused our analysis on mice made with *Stra8-Cre* and used the *Spo11-Cre* line to confirm our findings and to assess whether defects we observed originated during pre-meiotic or meiotic processes. *Hus1* deletion in the testis was confirmed by Southern blot detection of *Hus1* ^Δ*2,3*^, the null allele produced by CRE-mediated recombination (Figures S1C, S1D and S2D). As shown in Figure 1A and Figure S3B, Western blotting of whole testis lysates further confirmed that *Hus1* gene deletion using *Stra8-Cre* resulted in a drastic reduction in HUS1 protein level in the testis. Despite high genomic *Hus1* deletion efficiency, the reduction in HUS1 protein levels was more subtle in *Spo11-Cre Hus1* conditional knockout (CKO) mice, consistent with expectation that CRE expression from the *Spo11* promoter would result in HUS1 loss in a more restricted subset of cells and with delayed kinetics relative to that in mice with *Stra8-Cre* (Figure S3C). *Hus1* loss also led to a significant reduction in total RAD9 and RAD1 protein levels in testes from *Stra8-Cre Hus1* CKO animals (Figure S3A, S3B), indicating that the entire 9-1-1 complex was destabilized in the absence of HUS1.

### Conditional *Hus1* inactivation results in reduced testis size, reduced epididymal sperm count, and severely compromised fertility

In both the *Stra8-Cre* and *Spo11-Cre Hus1* CKO models, *Hus1* loss resulted in reduced adult testis size (Figure 1B, 1E). As expected, the phenotype was more severe in *Stra8-Cre Hus1* CKOs with earlier *Hus1* loss than *Spo11-Cre Hus1* CKOs. Testis weights were significantly reduced in *Stra8-Cre Hus1* CKO males as early as postnatal day 17 (Figure 1C), raising the possibility of roles for *Hus1* during early meiotic and/or pre-meiotic stages, including during spermatogonial DNA replication given the role of 9-1-1 during S-phase in somatic cells [2], [48]–[50]. *Spo11-Cre Hus1* CKO males, on the other hand, exhibited normal testis sizes at 17 and 28 days but significantly reduced testis weight in adults (Figure 1F; data not shown), consistent with meiotic defects beginning at the spermatocyte stage when the *Spo11* promoter is active. Due at least in part to greater fecundity in the FVB strain background, experimental mice were produced more readily using *Stra8-Cre* (FVB) compared to *Spo11-Cre* (129/B6) mice, and we therefore focused our further analysis of meiotic chromosome stability on the *Stra8-Cre Hus1* CKO model. As described below, our data indicate that the majority of meiotic phenotypes associated with *Hus1* inactivation are similar in both models.

The reduction in testis size in *Stra8-Cre* and *Spo11-Cre Hus1* conditional knockout animals was accompanied by significant reductions in the production of spermatozoa. The number of sperm in the caudal epididymis of 12-week old *Stra8-Cre* or *Spo11-Cre* conditional *Hus1* knockout males was reduced by approximately 10-fold and 2-fold, respectively (Figure 1D, 1G). To test sperm function in these mice, we mated *Stra8-Cre Hus1* conditional knockout males to wild-type female mice. While control matings produced 18 pregnancies and 154 viable pups, 49 matings with 5 different *Stra8-Cre Hus1* CKO males produced only 2 pregnancies and no viable pups (Table 1). Overall, we conclude that HUS1 is required for normal spermatogenesis and fertility.

**Table 1:** *Hus1* inactivation in the mouse testis results in severely reduced fertility.

	# males	# matings	# copulatory plugs	# pregnancies	Total viable pups
Controls	3	24	23	18	154
*Hus1* CKOs	5	49	44	2	0

### *Hus1* loss results in germ cell depletion

**Figure 2:**
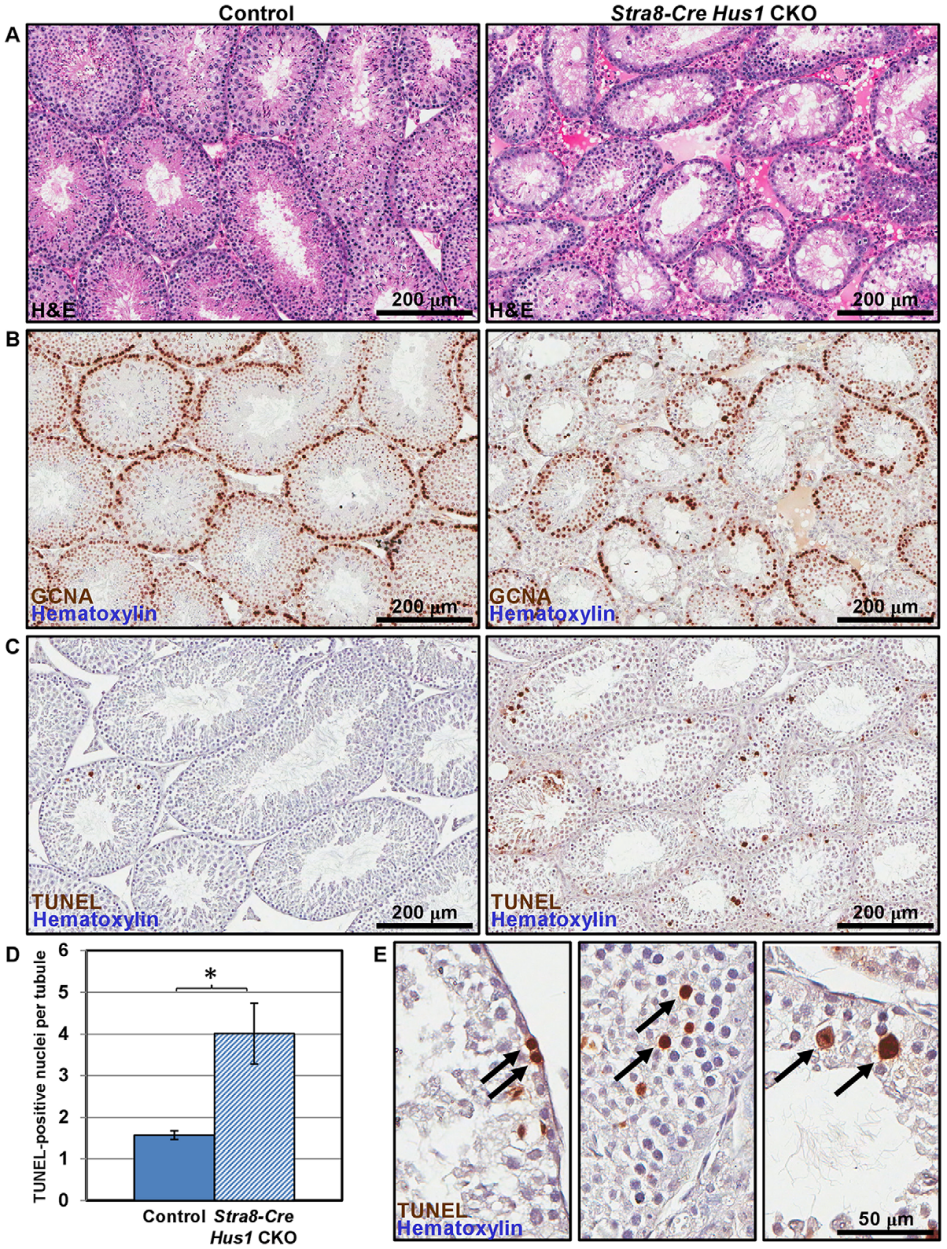
*Hus1* loss results in germ cell depletion. A. 100× images of H&E-stained histological sections from 12-week old control (left; *Cre*+ *Hus1^+/flox^*) and *Stra8-Cre Hus1* CKO (right) testes. B. GCNA1 staining of germ cells in control and *Stra8-Cre Hus1* CKO testes indicating germ cell loss. C. TUNEL staining of control and *Stra8-Cre Hus1* CKO adult testes indicating germ cell apoptosis. D. Quantification of TUNEL staining shown in C, shown as the mean ± SEM. Asterisk indicates statistically significant difference between *Hus1* CKO and control animals (p<0.05, Student's *t*-test). E. Higher magnification (400×) images of TUNEL staining in *Stra8-Cre Hus1* CKO adult testes. Arrows highlight TUNEL-positive cells that appeared to be pre-meiotic (left), mid-prophase I (middle), or at meiotic metaphase (right).

To determine the underlying cause of the fertility defects in *Hus1-*deficient mice, we analyzed testes from *Stra8-Cre Hus1* CKO and control animals. As shown in Figure 2A, *Stra8-Cre Hus1* CKO adult testes exhibited a marked decrease in tubule size and cellularity, with many tubules lacking spermatogenic cells within the lumen, and an abundance of both pyknotic nuclei as well as multinucleate spermatid giant cells. We additionally performed immunohistochemical staining with GCNA1 antibody to detect germ cells in testis sections. *Stra8-Cre Hus1* CKO testes exhibited significant loss of germ cells, including spermatogonia (Figure 2B). To further assess germ cell loss, we utilized TUNEL staining to detect fragmented DNA in *Stra8-Cre Hus1* CKO nuclei. *Hus1* mutant testes exhibited a significant increase in TUNEL-positive nuclei at both 17 days (Figure S4) and 12 weeks (Figure 2C–2E). Interestingly, a significant proportion of TUNEL-positive nuclei appeared to have progressed beyond pachytene stage to the first meiotic division (arrows, Figure 2E, right panel). 21% of all TUNEL-positive tubules contained apoptotic metaphase cells, although the majority of TUNEL-positive tubules (75%) contained apoptotic cells of a morphology consistent with zygotene/pachytene spermatocytes (Figure 2E, arrows, center panel). *Spo11-Cre Hus1* CKO animals, in which *Hus1* inactivation occurred later in germ cell development, also exhibited a decrease in testis cellularity and an increase in both multinucleate spermatid giant cells and TUNEL-positive cells (Figure S5). Altogether, these data indicate a requirement for *Hus1* and the 9-1-1 complex for germ cell maintenance and development.

### *Hus1* loss results in abnormal γH2AX localization and increased CHK1 phosphorylation

**Figure 3:**
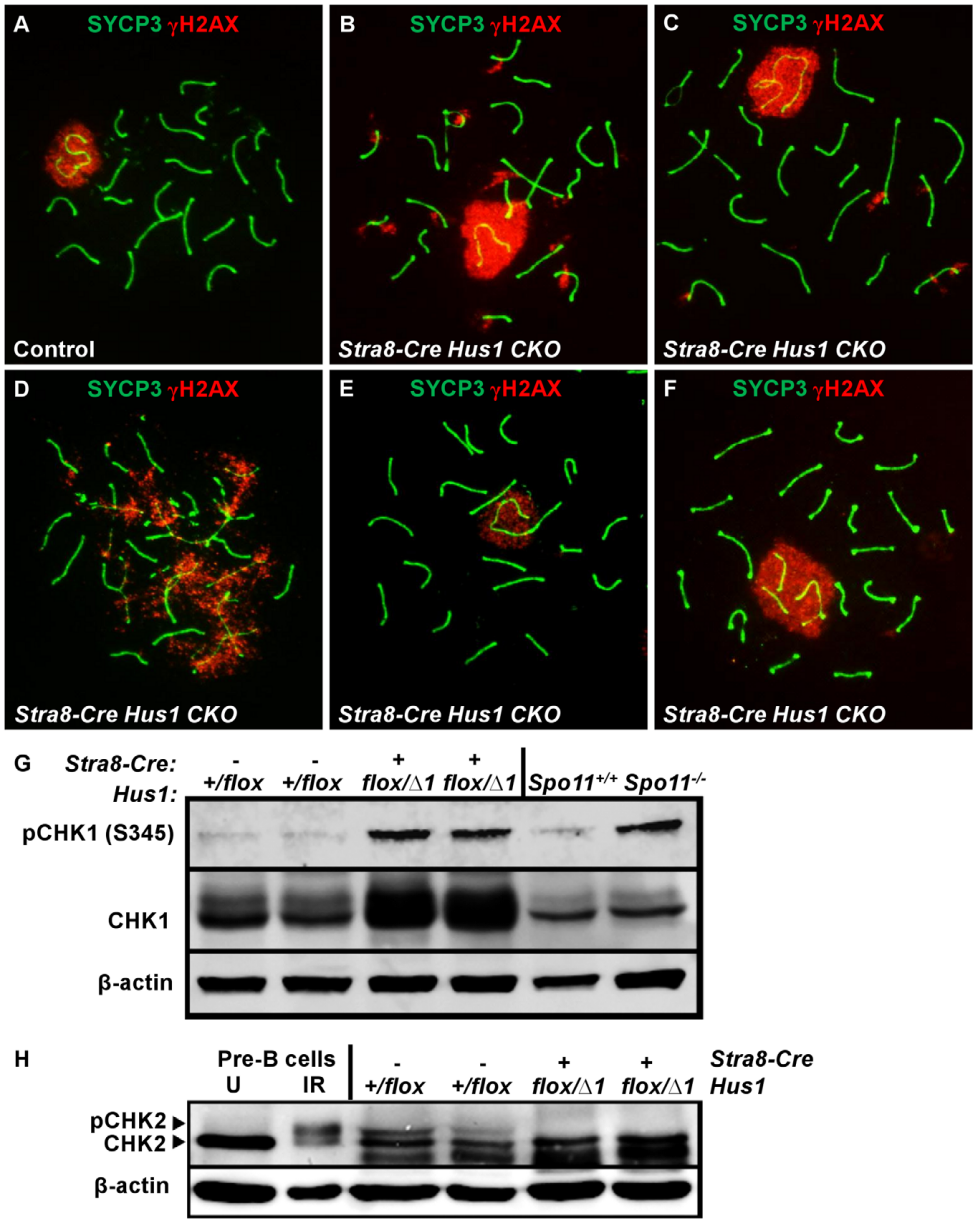
*Hus1* inactivation results in abnormal accumulation of γH2AX on autosomes, an extended sex body domain, inclusion of autosomes within the sex body, and X-autosome fusions. A–F. Immunofluorescence staining for γH2AX and SYCP3. γH2AX staining persisted in a perpendicular pattern on synapsed autosomes in pachytene (B) and diplotene stage (C), and in clouds surrounding unsynapsed chromosomal regions (D). The sex body domain marked by γH2AX was extended in *Stra8-Cre Hus1* CKOs (B,F), and often contained whole or partial autosomes (B,F). Some *Stra8-Cre Hus1* CKO nuclei exhibited apparent X chromosome-autosome end-to-end fusions (E). G,H. Western blot analysis of CHK1 (G) and CHK2 (H) in total testis extracts from mice of the indicated genotypes. In the CHK2 immunoblot, pre-B cells that were untreated (U) or treated with 5Gy ionizing radiation (IR) are included as controls. β-actin is shown as a loading control. In *Hus1* CKO samples, total CHK1 levels were increased on average 2.9-fold over controls, and phosphorylated CHK1 (S345) levels were increased 4.1-fold. CHK2 phosphorylation was not detected in the absence of *Hus1*.

Because the 9-1-1 complex protects chromosomal integrity in somatic cells, we hypothesized that germ cell loss in *Hus1* CKOs might be due to DNA damage accumulation. We prepared meiotic chromosome spreads and assessed the localization of γH2AX, the phosphorylated form of the histone variant H2AX, by indirect immunofluorescence. γH2AX marks DSBs as well as unsynapsed chromatin, and is phosphorylated by the ATM and ATR kinases [26], [29]. We found that γH2AX staining during leptotene and zygotene, when meiotic DSBs are generated and processed, was similar in *Hus1* CKOs and controls. Furthermore, γH2AX localized normally to the sex body domain in pachytene *Hus1* CKO spermatocytes (Figure 3), suggesting that ATR-dependent H2AX phosphorylation is independent of HUS1 as is also true during responses to replication stress [51]. However, in *Hus1* mutant spermatocytes, the sex body domain marked by γH2AX was enlarged and extended, and often encompassed parts of or entire autosomes (Figure 3B, 3C, 3F). 56% of mutant pachytene spermatocytes showed some type of sex body abnormality (compared to 13% of control nuclei), with 39% of *Stra8-Cre Hus1* CKO nuclei having sex body extensions or protrusions and 33% with autosome inclusion (compared to 4% and 9%, respectively, in control spermatocytes; Table 2). Similar phenotypes were also observed in *Spo11-Cre Hus1* CKO spermatocytes (Table 2). Autosomes included in the sex body domain often appeared synapsed (based on chromosome number and SYCP3 staining intensity), though asynapsed chromosomes and parts of chromosomes were also included in the sex body, as shown in Figure 3F. Additionally, a substantial number of meiotic nuclei harbored apparent end-to-end fusions between the X chromosome and an autosome (Figure 3E). In such cases, the X and Y chromosomes were synapsed at the pseudoautosomal region (PAR) and were contained within the sex body domain containing γH2AX, while the autosome remained outside of the sex body domain and was devoid of γH2AX. The frequency of X-autosome fusions was significantly increased in *Stra8-Cre Hus1* CKO pachytene spermatocytes, from 2% in controls to 12% in CKO mice (p<0.001; Table 2).

**Table 2:** Patterns of γH2AX localization in *Stra8-Cre* and *Spo11-Cre Hus1* CKO spermatocytes.

	Pachytene			
	Control	*Stra8-Cre Hus1* CKO	Control	*Spo11-Cre Hus1* CKO
N	255	252	115	114
Grossly normal	78%	34%***	71%	46%***
Autosomal γH2AX foci	15%	39%***	10%	30%***
Abnormal sex body	13%	56%***	24%	41%**
Extended sex body	4%	39%***	14%	31%**
Autosomes in sex body	9%	33%***	14%	29%**
X-autosome fusions	2%	12%***	3%	4%
XY paired	93%	84%**	88%	89%
Peripheral sex body	69%	56%**	78%	59%**

	Diplotene			
	Control	*Stra8-Cre Hus1* CKO	Control	*Spo11-Cre Hus1* CKO
N	106	69	53	36
Grossly normal	77%	36%***	89%	53%***
Autosomal γH2AX foci	4%	35%***	9%	17%
Extended sex body	6%	25%***	0%	0%
Autosomes in sex body	10%	19%	2%	0%
X-autosome fusions	5%	6%	0%	3%
SC ruptures	6%	36%***	2%	33%***
XY paired	73%	55%*	59%	56%
Peripheral sex body	73%	57%*	76%	69%

* p<0.05,

** p<0.01,

*** p<0.001,

*Hus1* CKO relative to control; z-test of sample proportions.

Extended sex body domains marked by γH2AX persisted in diplotene-stage *Stra8-Cre Hus1* CKOs (25% of *Hus1* CKO nuclei versus 6% of controls; Table 2), indicating that these cells did not arrest at the pachytene/diplotene transition despite chromatin abnormalities. Additionally, the sex body domain in both pachytene and diplotene spermatocytes was located at the nuclear periphery less often in both *Stra8-Cre* and *Spo11-Cre Hus1* CKOs than in controls (Table 2), indicating a perturbation in the normal compartmentalization or localization of the sex chromatin. Although we cannot entirely rule out the possibility that the 9-1-1 complex directly promotes sex body maintenance through stimulation of ATR activity or other roles, we favor the idea that defects in maintenance of sex body integrity following *Hus1* loss could instead be related to aberrant responses to meiotic DSBs. In cases where autosomes were asynapsed, clouds of γH2AX surrounded the unsynapsed regions (Figure 3D), supporting the idea that ATR-mediated responses to unsynapsed chromatin remain intact despite *Hus1* loss. Consistent with the idea that expanded sex body domains marked by γH2AX reflect upregulation of ATR kinase activity, we also observed that *Hus1* loss was associated with increased levels of both total and phosphorylated CHK1 (Figure 3G and Figure S3), the latter being an established indicator of ATR activity in somatic cells [52]. Increased phosphorylated CHK1 was also observed in extracts prepared from *Spo11*-deficient testes (Figure 3G), which have severe defects in chromosome synapsis.

A significant percentage of pachytene *Stra8-Cre Hus1* CKO nuclei exhibited γH2AX staining on fully synapsed autosomes (39% versus 15%, p<0.001; Table 2), generally in a burst-like pattern perpendicular to the synaptonemal complex, indicative of unrepaired DSBs (Figure 3B). This eruption-like pattern of γH2AX staining on *Hus1* CKO cores is consistent with previously described γH2AX L-foci [53], which are proposed to be sites of delayed or unregulated DSB repair events, and have been observed in several other meiotic mutants with break repair defects [54]–[56]. A similar pattern of γH2AX staining was observed in *Spo11-Cre-* and *Stra8-Cre*-driven *Hus1* CKO mice (Table 2), suggesting that many of the persistent breaks originate during meiosis and are not due exclusively to aberrant pre-meiotic replication in the absence of *Hus1*. The autosomal γH2AX staining in *Hus1* CKOs persisted into diplotene (Figure 3C), with 35% of *Hus1* mutant nuclei containing bursts of autosomal γH2AX at this stage compared to 4% of control nuclei (Table 2). Persistence of these γH2AX foci into diplotene indicates that homologous chromosomes began to desynapse despite incomplete DSB repair, and that these aberrant spermatocytes did not arrest in the pachytene stage in the absence of *Hus1*. Consistent with the possibility of impaired DSB-induced DNA damage checkpoint signaling following *Hus1* loss, we observed that the phosphorylated form of the DSB-responsive checkpoint kinase CHK2, which was evident in extracts from control testes, was absent in those from adult *Hus1* CKO mice (Figure 3H and Figure S3B).

### RAD51 foci persist in *Hus1*-deficient spermatocytes, primarily at sites lacking MLH1

**Figure 4:**
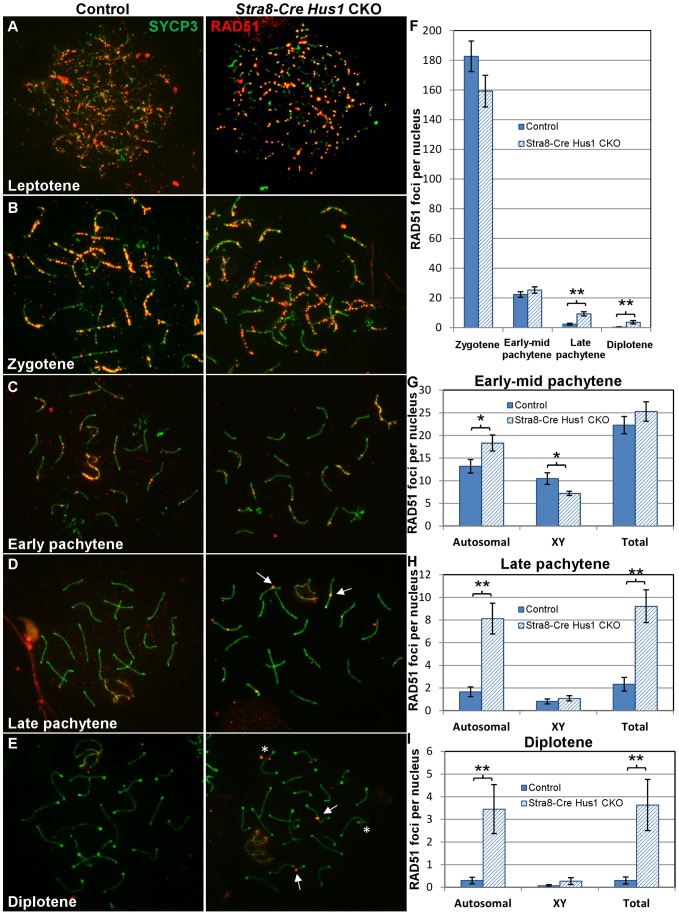
RAD51 foci persist in late pachytene and diplotene spermatocytes in the absence of *Hus1*. A–E. Immunofluorescence staining for RAD51/DMC1 from zygotene through diplotene stage of meiosis in control (*Cre-*negative *Hus1^flox/^* ^Δ*1*^) and *Stra8-Cre Hus1* CKO males. Arrows indicate persistent foci, and asterisks indicate symmetrical foci located on either side of chromosomal cores. F–I. Quantification of RAD51 foci at the indicated stages. ** indicates p<0.01; * indicates p<0.05.

Since increased numbers of γH2AX foci were present on *Hus1* CKO meiotic chromosomes, we further investigated whether the RAD51 strand exchange protein accumulated normally on *Hus1*-deficient chromosomes. During the early stages of meiotic DSB repair in zygotene and early pachytene spermatocytes, RAD51 properly localized to punctate structures along meiotic chromosome cores in the absence of *Hus1* (Figure 4A). By late pachytene, when most RAD51 foci are normally lost from autosomal and XY chromosome cores, all *Hus1* CKO spermatocytes exhibited persistent localization of RAD51 to the autosomes as well as the X chromosome, indicative of a DSB repair defect (Figure 4C). These foci persisted into diplotene as well (Figure 4D), demonstrating that nuclei with unrepaired DSBs were also able to progress beyond the pachytene stage. RAD51 focus numbers were quantified at each of these stages (Figure 4F–4I), and the number of foci in *Hus1* CKO nuclei was significantly increased in late pachytene and diplotene. While the number of persistent RAD51 foci per nucleus was modest, the total number of nuclei with at least one RAD51 focus was high in *Hus1* CKOs, at 100% of *Hus1* CKO nuclei versus 52% of controls in late pachytene, and 73% of *Hus1* CKOs versus 17% of controls in diplotene (Figure 4). Further, the reduction in the frequency of cells with RAD51 foci from 100% to 73% of *Hus1* CKO nuclei from late pachytene to diplotene could indicate that *Hus1* mutants can repair DSBs, albeit inefficiently in some cases, such that repair is significantly delayed. We conclude that HUS1 is critical for efficient completion of DNA repair at a subset of meiotic DSBs.

To determine whether *Hus1* loss alters later stages in DSB repair such as crossover formation, we assessed MLH1 localization in pachytene spermatocytes. The mismatch repair complex MLH1-MLH3 is required for the formation of Class I crossover events, which account for approximately 90% of all meiotic crossovers in mice [57], [58]. Mouse and human RAD9 proteins have been shown to interact with MLH1 [21], although it is unclear whether this interaction is relevant for meiotic crossover recombination or whether it is limited primarily to post-replicative DNA mismatch repair. As shown in Figure S6A, the total number of MLH1 foci per nucleus was not significantly different in *Hus1* CKO cells relative to controls, with one to two MLH1 foci per homolog pair. *Hus1* CKOs exhibited a mean of 25.4±0.3 MLH1 foci per nucleus, compared to 25.9±0.3 in controls (Figure S6B), suggesting that *Hus1* loss does not significantly impair crossover formation. However, a general impairment of meiotic DSB repair in the absence of functional 9-1-1 complex might manifest as only a modest reduction in the number of MLH1 foci per nucleus, as 10% of crossovers are not dependent on MLH1-MLH3 and the majority of meiotic DSBs are repaired as noncrossovers [59]. To determine whether the persistent RAD51 foci observed in *Hus1* CKOs were primarily associated with noncrossover or crossover events, we assessed the relative localization of RAD51 and MLH1. As shown in Figure S6C, the majority of persistent RAD51 foci in late pachytene were exclusive of MLH1, though an occasional single RAD51 focus localized at or adjacent to an MLH1 focus. These data indicate that while some meiotic DSBs are not repaired efficiently in the absence of *Hus1*, these unrepaired breaks consist primarily of MLH1-independent repair events (most likely noncrossovers) but can include MLH1-dependent crossovers.

Since a significant proportion of TUNEL-positive *Hus1* CKO germ cells appeared to be in meiotic metaphase, it remained possible that *Hus1* CKOs might be deficient in crossover formation, despite the normal complement of MLH1 foci we observed. Thus, we prepared meiotic diakinesis chromosome spreads from control and *Hus1* CKO testes to assess chromosome integrity at the end of prophase I, when the SC has disintegrated and chiasmata are first evident. As shown in Figure S6D–S6E, the majority of nuclei had 20 bivalents, indicating that each homolog pair received at least the one obligate crossover necessary for proper chromosome segregation. Taken together, these results indicate that while HUS1 is critical for repair of a subset of meiotic DSBs, it appears to be dispensable for crossover formation.

### *Hus1* inactivation results in meiotic chromosome defects

**Figure 5:**
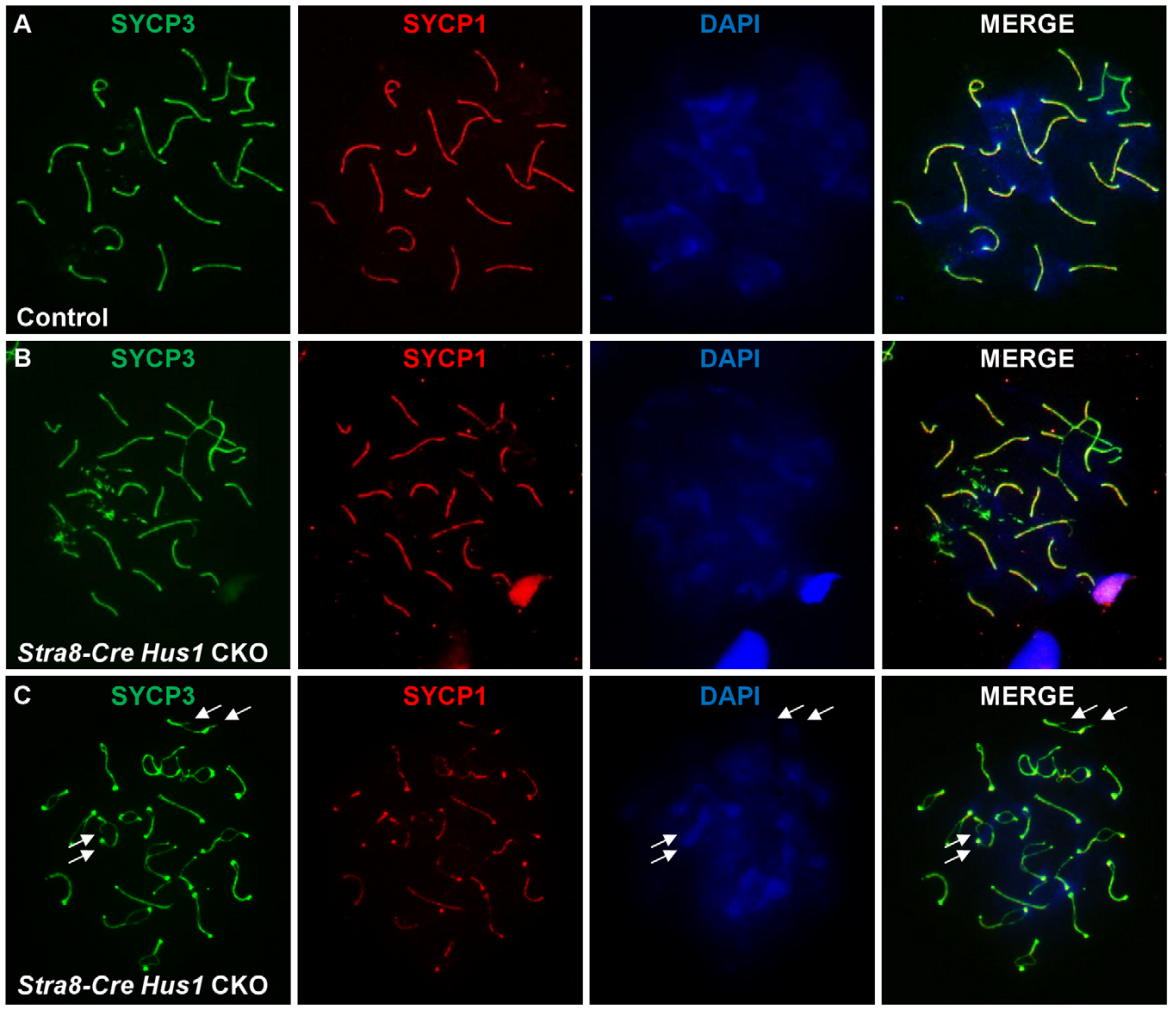
Conditional *Hus1* knockout meiotic chromosomes display synapsis defects and ruptures in the synaptonemal complex. Meiotic chromosome spreads from control (*Cre-*negative *Hus1^flox/^* ^Δ*1*^) and *Stra8-Cre Hus1* CKO mice were stained for SYCP1 and SYCP3, as well as with DAPI. A. Normal synapsis of pachytene chromosomes in control males, as indicated by SYCP1 and SYCP3 immunofluorescence. B. Chromosomal asynapsis in pachytene-stage *Stra8-Cre Hus1* CKO nuclei, with unsynapsed chromosomal regions devoid of SYCP1. C. Diplotene *Stra8-Cre Hus1* CKO chromosomes with ruptures in the SC lateral elements, as indicated by arrows.

To investigate additional causes for germ cell loss in *Hus1* CKOs, we assessed the localization of various meiotic markers on chromosome spreads from control and *Stra8-Cre Hus1* CKO males. Meiotic chromosome synapsis appeared normal in most *Hus1* mutant nuclei, with colocalization of the synaptonemal complex proteins SYCP1 and SYCP3 in pachytene spermatocytes and the presence of 19 pairs of fully synapsed homologs in addition to the X and Y chromosomes paired at the pseudoautosomal region. However, as shown in Figure 5B, a significant number of *Stra8-Cre Hus1* CKO nuclei exhibited synapsis defects, usually involving the X chromosome (14% of *Hus1* CKOs versus 3% of controls; N = 161 and 185, respectively; p<0.001). Notably, *Hus1* CKOs also frequently displayed ruptures in the synaptonemal complex during diplotene (36% of *Stra8-Cre Hus1* CKOs and 33% of *Spo11-Cre Hus1* CKOs compared to 2–6% of controls; Table 2). In these cases, there were clear discontinuities in synaptonemal complex protein staining and the ends of the broken SC were spatially separated (Figure 5C, arrows), indicating either a defect in SC integrity or breakage of chromosomal DNA in the absence of HUS1.

### TOPBP1 localization and RNA Pol II exclusion from the sex body remain intact in the absence of *Hus1*

**Figure 6:**
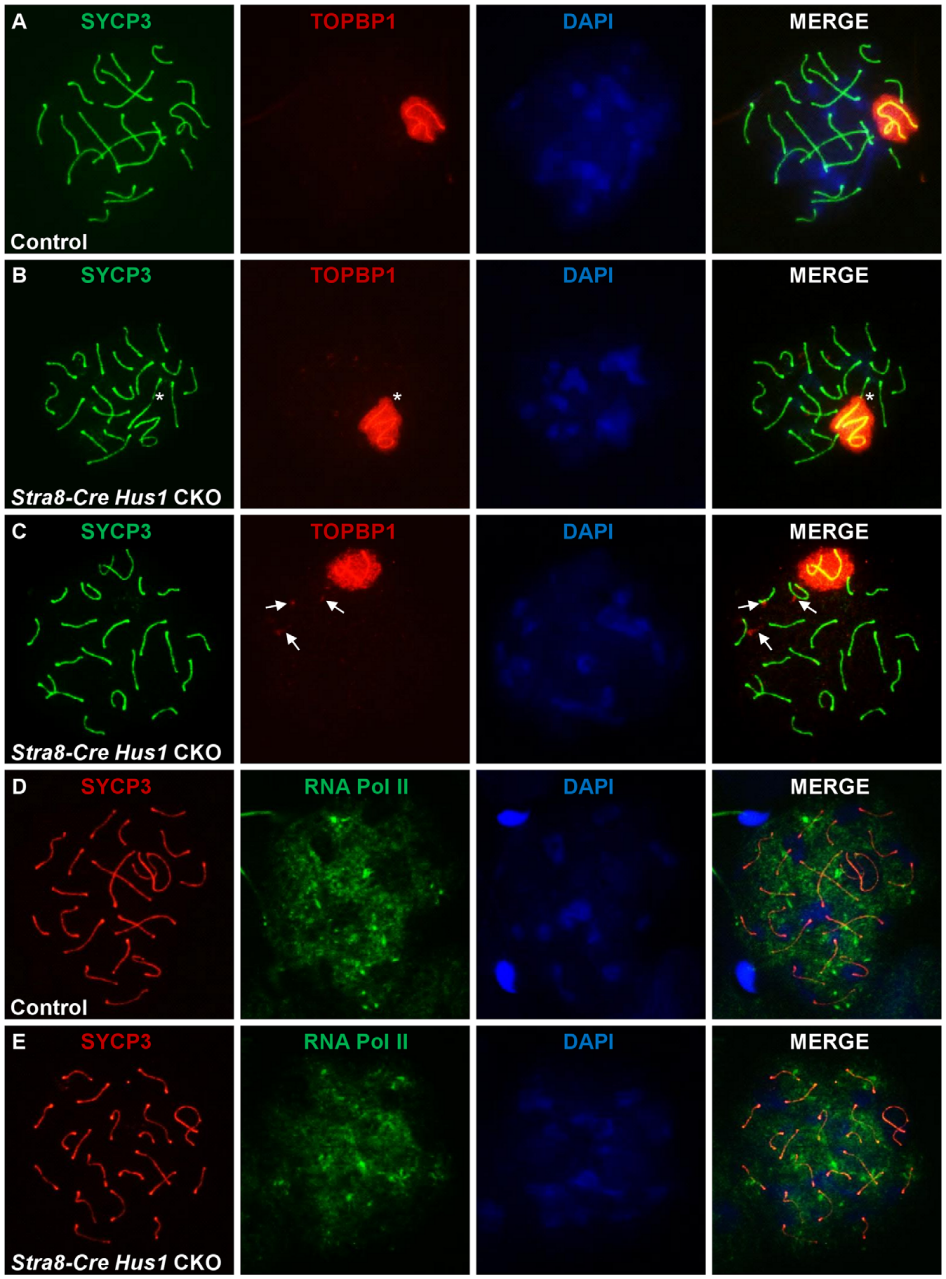
TOPBP1 localization to and RNA Pol II exclusion from the sex body domain remain unperturbed following *Hus1* inactivation. A–E. Immunofluorescence staining for TOPBP1 (A–C) and RNA Polymerase II (D–E) in control (*Cre*-negative *Hus1^flox/^* ^Δ*1*^) and *Stra8-Cre Hus1* CKO pachytene spermatocytes. The asterisk in B indicates an autosome partially included within the sex body domain and coated with TOPBP1. Arrows in C indicate abnormal TOPBP1 staining in a perpendicular pattern on autosomes in pachytene *Hus1* CKOs.

During meiosis, TOPBP1 normally colocalizes with ATR at sites of unsynapsed chromatin, including unsynapsed autosomes in early spermatocytes as well as the XY chromatin throughout prophase I [24], [60]. In mammalian somatic cells, the best characterized function of 9-1-1 is to recruit TOPBP1 to sites of DNA damage, where it physically interacts with and stimulates the kinase activity of ATR [61]–[63]. Therefore, we next tested whether *Hus1* loss affected the meiotic localization of TOPBP1. In contrast to the 9-1-1-dependent mechanism elucidated in somatic cells, TOPBP1 localization to the asynapsed sex chromatin was unperturbed in *Hus1* CKO mice (Figure 6). Additionally, TOPBP1 localized to autosomes that resided within the sex body domain (Figure 6B, asterisk), and to perpendicular foci on some autosomes in *Stra8-Cre Hus1* CKOs (Figure 6C) similar to the γH2AX eruptions described above, indicating that HUS1 loss does not preclude assembly of TOPBP1 on chromatin and results in TOPBP1 localization to abnormal autosomes as well as to unsynapsed sex chromatin.

Because the downstream consequence of ATR action at asynapsed meiotic chromatin is meiotic silencing [30], [32], we also assessed the localization of RNA Polymerase II, which indicates sites of active transcription. Consistent with the apparently normal TOPBP1 localization following *Hus1* deletion in germ cells, RNA Pol II immunofluorescence staining revealed no difference in transcriptional silencing of the XY chromatin, as indicated by a similar lack of RNA Pol II signal in the sex body domain of cells from both *Hus1* CKO and control mice (Figure 6D, 6E). Together, these results indicate that despite the absence of the HUS1 component of the 9-1-1 complex in *Hus1* CKO spermatocytes, TOPBP1- and ATR-dependent responses to unsynapsed chromatin remained intact. These findings raise the possibility that ATR activation in response to at least some signals during mammalian meiosis, such as unsynapsed chromatin, may occur through a distinct mechanism independent of the canonical 9-1-1 complex.

### RAD9 localizes to DSB sites on meiotic chromosomes during prophase I

**Figure 7:**
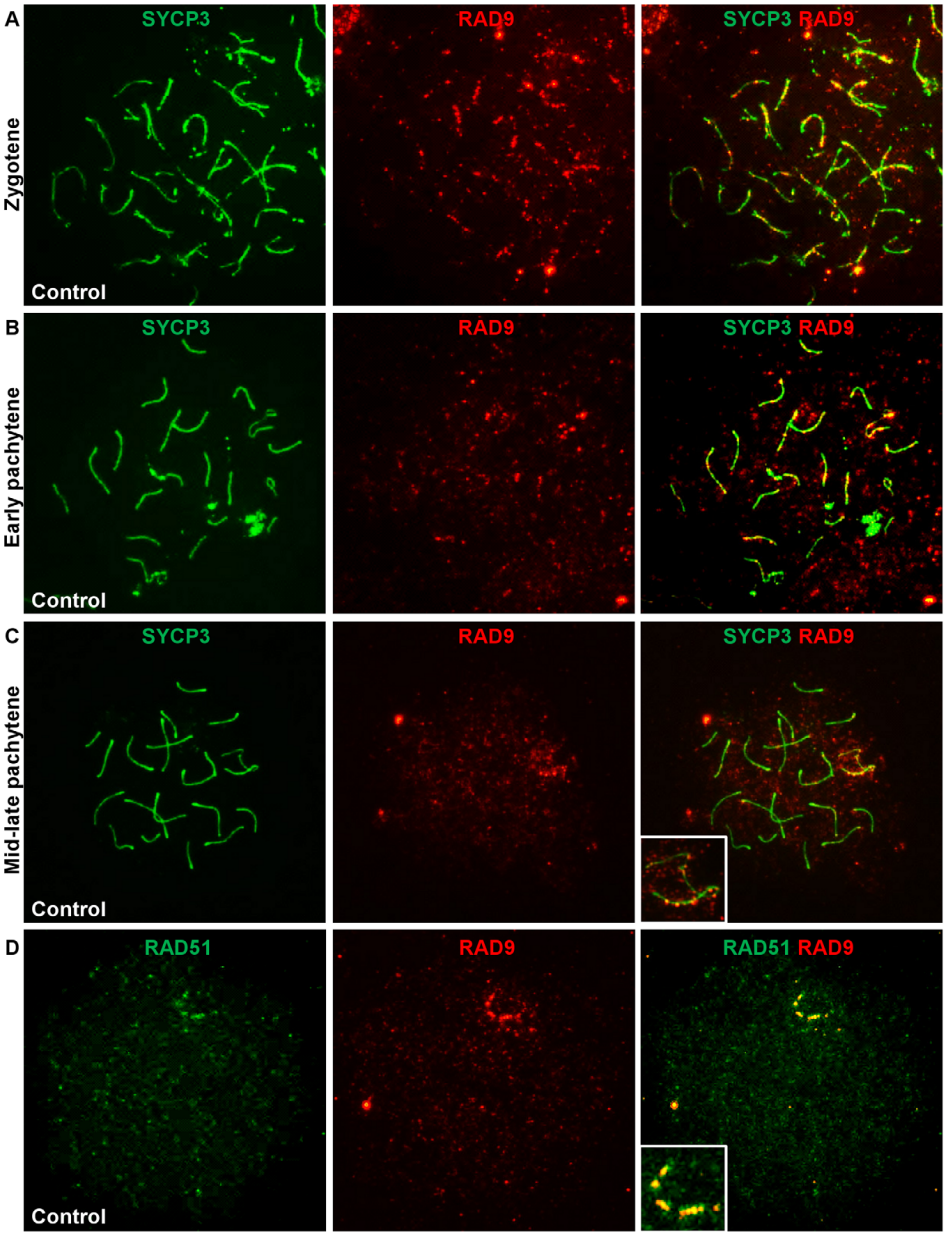
RAD9 localizes to meiotic chromosomes during early prophase I and colocalizes with a subset of RAD51 foci. A–C. Meiotic chromosome spreads from control animals were stained for RAD9 and SYCP3. In wild-type adult males, RAD9 localized along the synaptonemal complex of synapsed and unsynapsed chromosomes during zygotene (A) and pachytene (B, C). RAD9 localized to autosomes and the sex chromosomes in early pachytene (B), was confined primarily to the X chromosome by mid-late pachytene (C), and was absent by diplotene. D. Meiotic chromosome spreads from control animals were stained for RAD9 and RAD51. RAD9 colocalized with a subset of RAD51 foci, particularly along the X chromosome in pachytene-like nuclei.

Since *Hus1* disruption resulted in chromosomal defects without affecting TOPBP1 localization, we next analyzed RAD9 localization to meiotic chromosomes to gain insights into the normal functions of 9-1-1. As shown in Figure 7A–7C, RAD9 localized to meiotic chromosome cores during prophase I, with many foci on both unsynapsed and synapsed chromosomes during zygotene stage, fewer autosomal foci and prominent X chromosome foci in early pachytene, and even fewer foci by mid- to late-pachytene stage when most staining was confined to bright foci along the X chromosome core. By diplotene stage, RAD9 foci were not detectable on meiotic chromosomes. Thus, the RAD9 subunit of 9-1-1 is appropriately positioned for a potential role in genome maintenance during meiosis.

The pattern and timing of the RAD9 signal on meiotic chromosomes was reminiscent of that of the DSB repair factor RAD51, particularly with respect to its presence at late-persisting breaks along the X chromosome core. To determine whether RAD9 colocalized with RAD51, we simultaneously stained meiotic chromosomes for RAD9 and RAD51. In pachytene-like nuclei, the majority of RAD9 foci colocalized with RAD51 foci, most notably along what is presumably the X chromosome axial element (Figure 7D). Thus, RAD9 appears to localize to DSBs undergoing homologous recombinational repair. To further assess RAD9 function in DSB repair, we also analyzed RAD9 localization in *Spo11^−/−^* mutants [64], which lack meiotic DSBs, and *Dmc1^−/−^* mutants [65], which harbor persistent unrepaired DSBs. As shown in Figure S7, RAD9 staining was much reduced in the absence of meiotic DSBs and elevated in the presence of increased DSBs. Thus, RAD9, and likely its partner HUS1 (see below), function together at meiotic DSBs at a stage overlapping with RAD51/DMC1.

### The distribution of RAD9 and RAD1 on meiotic prophase I chromosomes is only partially overlapping

**Figure 8:**
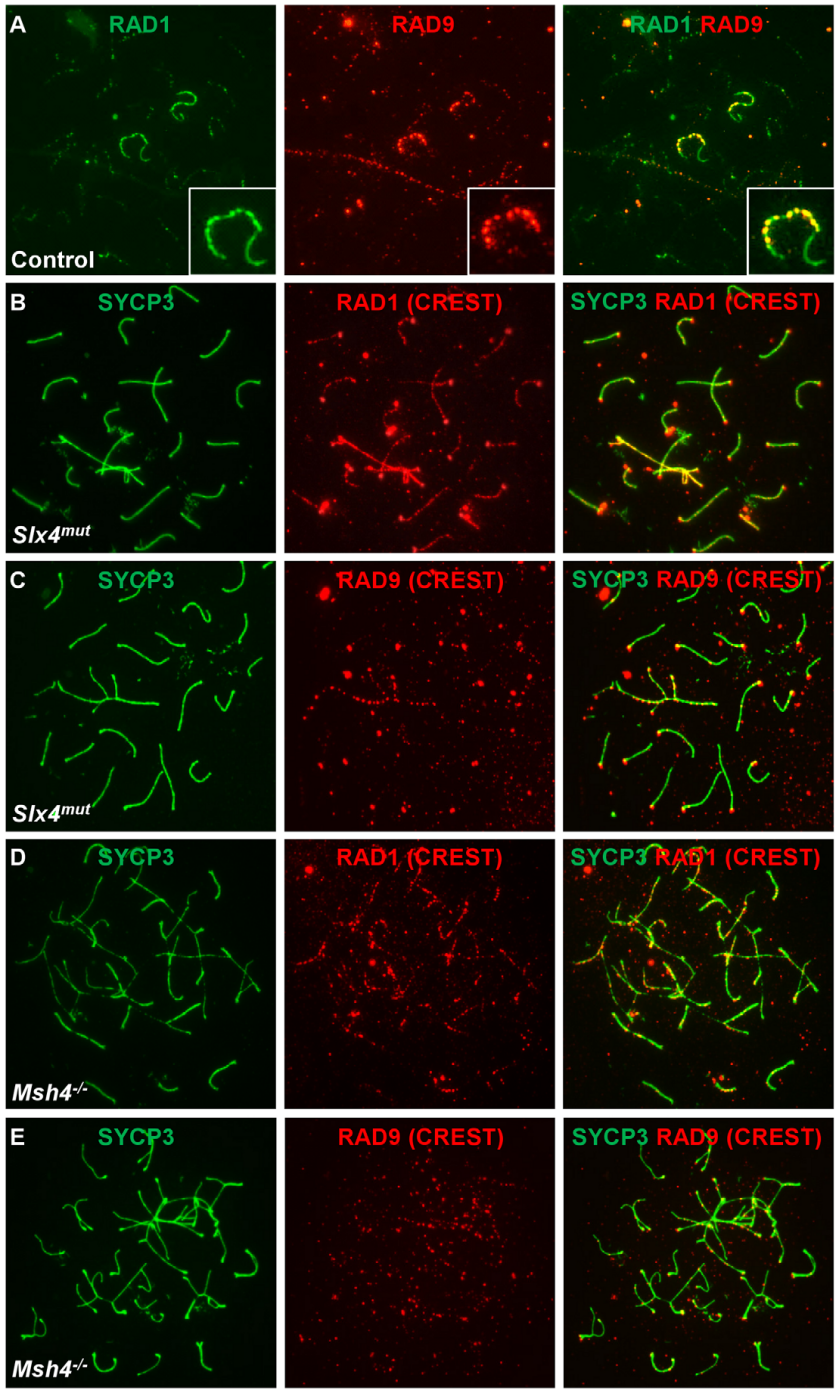
RAD9 and RAD1 localize in overlapping yet distinct patterns on meiotic chromosomes. A. Meiotic chromosome spreads from control animals were stained for RAD9 and RAD1. RAD9 colocalized with a subset of RAD1 foci in control spermatocytes. A region containing one X-Y pair is shown at higher magnification. B–E. Meiotic chromosome spreads from *Slx4^mut/mut^* (B, C) or *Msh4^−/−^* (D, E) mice were stained for SYCP3 and RAD1 (B, D) or RAD9 (C, E). RAD1 localized more continuously along sex chromosome cores and autosomes and to asynaptic sites in *Slx4* and *Msh4* mutants, whereas RAD9 formed fewer, more punctate foci along asynapsed chromosome cores. Chromosome spreads in panels B–E were additionally stained using human CREST serum, which marks centromeric regions. CREST signal is detected in the red channel (middle column) and in the merged images.

The RAD1 subunit of 9-1-1 was previously determined to localize to meiotic chromosomes, where RAD1 foci outnumber RAD51 foci and nearly continuously coat pachytene chromosome cores, especially along the unsynapsed X and Y [41]. To reconcile this staining with our observations of fewer, more punctate RAD9 foci, we assessed the colocalization of RAD1 and RAD9 subunits in control nuclei. These experiments revealed that RAD1 had a broader distribution than RAD9, with RAD1 foci present along the entire unsynapsed regions of the X and Y while RAD9 was detected primarily in bright foci along the X. Overall, only 23% of RAD1 foci colocalized with RAD9 (16% of autosomal and 46% of XY RAD1 foci colocalized with RAD9). A larger percentage (64%) of RAD9 foci colocalized with RAD1, particularly on the sex chromosomes (52% of autosomal and 86% of XY RAD9 foci colocalized with RAD1). We next assessed both RAD1 and RAD9 localization on abnormal chromosomes in *Slx4^mut/mut^* and *Msh4^−/−^* mutant spermatocytes, which exhibit asynapsis as well as inclusion of autosomes within the sex body domain [55], [66]. Consistent with the co-staining results, RAD1 localized along synapsed autosomal cores and to a greater extent along asynapsed autosomal and XY cores (Figure 8B, 8D). RAD9 on the other hand localized in a more punctate pattern along chromosome cores in the sex body domain and on asynapsed autosomes (Figure 8C, 8E). Together, these results indicate that RAD1 and RAD9 partially colocalize along abnormal chromosomes and the sex chromosome cores, and that a subset of RAD1-containing sites lack RAD9.

### *Hus1* loss differentially affects the localization of RAD9 and RAD1 on meiotic chromosomes

**Figure 9:**
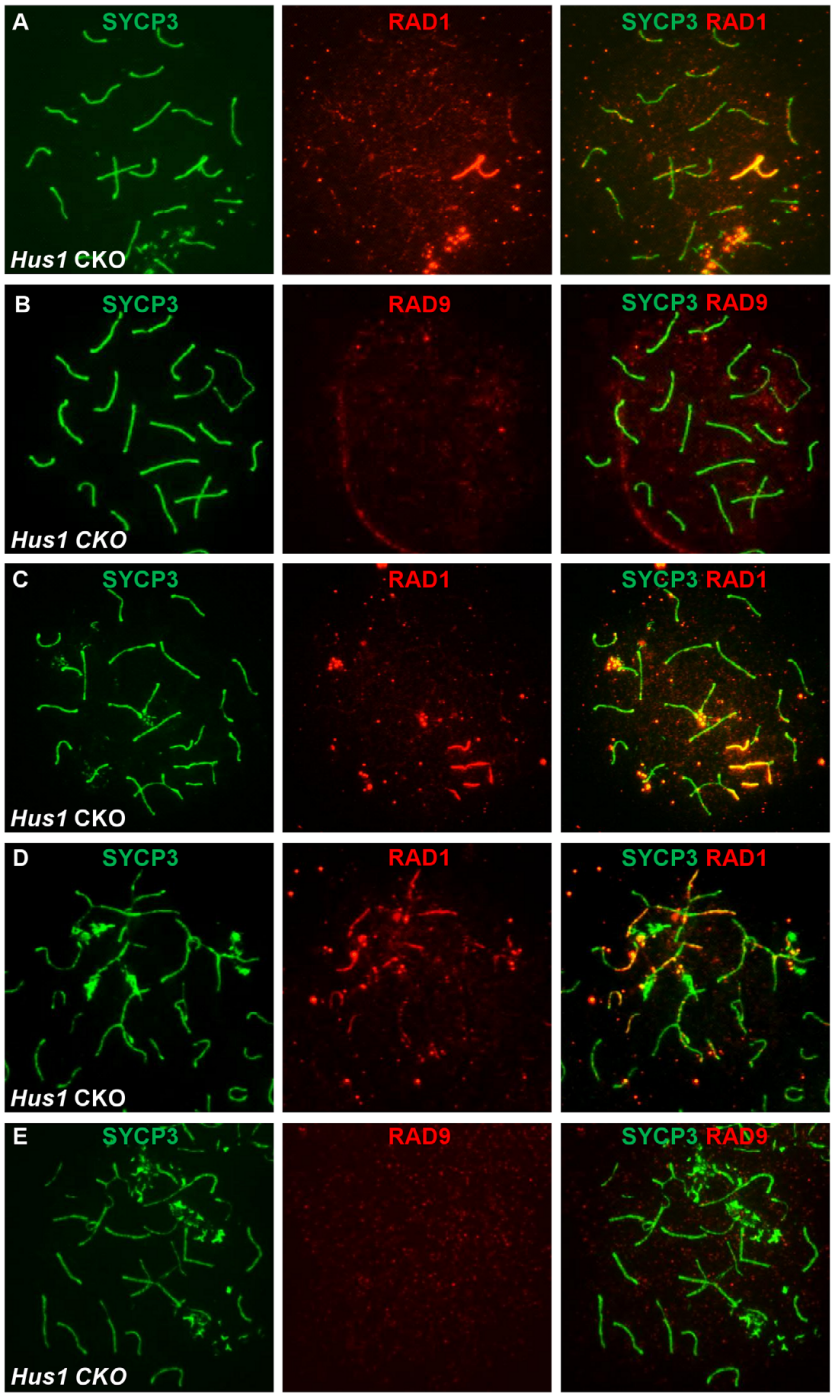
RAD1 and RAD9 localization to meiotic chromosomes is differentially affected by *Hus1* loss. Meiotic chromosome spreads from *Stra8-Cre Hus1* CKO mice were stained for SYCP3 and RAD1 (A, C, D) or RAD9 (B, E). A,C,D. RAD1 continued to localize to sex chromosomes and aberrant meiotic chromosomes in the absence of *Hus1*. Most notably, RAD1 localization to asynapsed autosomes and chromosomes with the sex body domain persisted in cells from *Hus1* CKO mice. By contrast, RAD9 localization to both normal and aberrant chromosome structures was abolished following *Hus1* loss (B,E).

Prompted by the differences in RAD1 and RAD9 localization described above, we next assessed localization of these 9-1-1 subunits in *Hus1* CKO spermatocytes. Since the chromatin association and nuclear localization of the 9-1-1 complex in somatic cells requires all three subunits [48], we expected RAD9 and RAD1 to be depleted from chromosomes in the absence of HUS1. However, the more extensive RAD1 staining observed in normal and aberrant spermatocytes suggested a possible separation of functions for the individual subunits. Remarkably, bright RAD1 staining of XY cores as well as punctate staining of autosomes persisted upon *Hus1* deletion (Figure 9A). By contrast, RAD9 foci were undetectable in nuclei from *Hus1* CKO mice (Figure 9B). Whereas RAD9 staining was readily detected in all control nuclei, approximately 99% of *Stra8-Cre Hus1* CKO pachytene nuclei lacked RAD9 foci. The continued presence of RAD1 on meiotic chromosomes following *Hus1* loss despite the significant reduction in total RAD1 levels (Figure S3) may indicate that a fraction of RAD1 protein participates in chromatin-associated complexes that remain stable independently of HUS1, while the remaining pool of RAD1 is unstable in the absence of HUS1.

The occasional occurrence of asynapsed homologs and the presence of autosomes within the sex body domain of pachytene *Hus1* CKO cells additionally allowed for evaluation of the dependency on *Hus1* for RAD9 and RAD1 recruitment to abnormal structures during meiosis. Similar to our observation that the localization of TOPBP1 was unperturbed in *Hus1* CKOs, RAD1 clearly localized to autosomes within the vicinity of the sex body domain (Figure 9C) and to asynapsed autosomes (Figure 9D) in the absence of *Hus1*. The localization of RAD9 on the other hand appeared strictly *Hus1*-dependent, as RAD9 was not detected on asynapsed chromosomes or sex chromosome cores in *Hus1* CKOs (Figure 9E), although it did localize to such structures in *Hus1*-expressing cells (Figure 7 and Figure 8). Together with the results described above, these data indicate that RAD9 and RAD1 have overlapping *Hus1-*dependent functions that intersect with RAD51, and that RAD1 has additional meiotic functions that may involve TOPBP1 but are independent of its traditional binding partners, HUS1 and RAD9.

## Discussion

In order to examine the role of the 9-1-1 complex during mammalian meiosis, we analyzed mice with germ cell-specific *Hus1* deletion and evaluated the localization of the RAD9 and RAD1 subunits to normal and aberrant meiotic chromosomes. Most notably, we report that the RAD9 and RAD1 subunits of the 9-1-1 complex exhibit overlapping yet distinct localization patterns along mammalian meiotic chromosomes and are differentially affected by loss of the HUS1 subunit. RAD1 forms more foci along chromosome cores than RAD9 (this study) or RAD51 [41], and we show that it preferentially localizes to the axial elements of asynapsed chromosomes or autosomes included within the sex body domain in a manner that is independent of *Hus1*. Previous electron microscopy analysis indicated that while RAD1 localizes to meiotic chromosomes cores, it does not colocalize with DMC1 [41], which is consistent with our finding of RAD1 at asynaptic sites. We show here that only a subset of RAD1 foci colocalize with RAD9, raising the possibility that RAD1 is present both at DSBs, as part of the canonical 9-1-1 complex, and at other chromosomal sites.

We propose a model in which meiotic genome maintenance involves several distinct checkpoint clamp complexes, some of which function in DSB repair, and others of which carry out separate functions, possibly including ATR activation through TOPBP1 in response to asynapsis. Of particular note are the previous findings that the mouse and human genomes contain paralogs of RAD9 and HUS1, termed RAD9B and HUS1B, respectively, that are highly expressed in the testis [67], [68]. Given that RAD1 localized to some chromosomal sites that lacked RAD9 and did so following genetic ablation of *Hus1*, it is tempting to speculate that RAD9B and HUS1B associate with RAD1 to form an alternative 9-1-1-like heterotrimer, although it remains possible that RAD1 localizes to chromosomes on its own or in conjunction with other cofactors. In this regard, it is worth noting that a substantial portion of RAD1 protein in cultured human cells exists in monomeric form, independent of the heterotrimeric 9-1-1 complex [69]. Overall, our results are consistent with formation of the canonical 9-1-1 complex composed of RAD9, RAD1, and HUS1 at meiotic DSBs, as well as with RAD1 assembling on chromosomes independently of HUS1 and RAD9 in patterns overlapping with TOPBP1 and ATR.

Despite a well-established role for 9-1-1 in ATR activation in somatic cells, we observed unperturbed localization of the 9-1-1-interacting, ATR-activating TOPBP1 protein and similarly no change in RNA Polymerase II exclusion from the XY body domain in *Hus1* CKO spermatocytes. These findings indicate that ATR-dependent responses to unsynapsed chromatin, including meiotic silencing via MSUC and MSCI, do not require HUS1, which is in distinct contrast to the canonical model of mammalian ATR activation in which 9-1-1-mediated TOPBP1 recruitment to damaged DNA allows TOPBP1 to contact and activate ATR. Since RAD1 localization to asynapsed meiotic cores was also unperturbed in the absence of *Hus1*, it is possible that a RAD1-containing complex functions along with TOPBP1 to activate ATR at such sites, in a manner that may be at least in part distinct from established mechanisms for 9-1-1 loading and function at DNA damage sites. The meiosis-specific HORMAD1 and HORMAD2 proteins are required for ATR recruitment to sites of unsynapsed chromatin [33], [70], and together with BRCA1, which is also required for ATR recruitment [26], these proteins could provide an alternative mechanism for ATR activation that is independent of 9-1-1 and typical substrates for 9-1-1 loading. We favor the idea that alternative complexes involving RAD1 and possibly RAD9B and HUS1B might engage in a 9-1-1-like interaction with TOPBP1. It remains to be determined whether the recruitment of such a complex to asynaptic sites is facilitated by particular DNA structures or binding partners that would not attract conventional 9-1-1 complexes.

Conditional *Hus1* deletion in germ cells resulted in numerous chromosomal defects, including asynapsis, unrepaired meiotic DSBs, X-autosome end-to-end fusions, autosomes incorporated into the sex body domain (which presumably are inappropriately transcriptionally silenced), and SC ruptures. We propose that some of these defects (asynapsed chromosomes, X-autosome fusions, and autosomes incorporated into the sex body domain) lead to loss of cells during the pachytene stage in *Hus1* CKOs, consistent with an absence of cells with such abnormalities in diplotene (Table 2), whereas other defects (unrepaired DSBs, extended sex body domains, and SC ruptures; Table 2) either may not be actively monitored or may normally require HUS1 for checkpoint-dependent clearance and therefore persist beyond pachytene into diplotene in *Stra8-Cre Hus1* CKOs. The latter set of phenotypes was less prominent in *Spo11-Cre Hus1* CKO mice, with fewer defective cells persisting into diplotene, suggesting that HUS1-dependent checkpoint monitoring may be an earlier function that is less affected by mid-prophase I loss of *Hus1*. Notably, SC ruptures were still prominent in diplotene cells of both *Stra8-Cre* and *Spo11-Cre Hus1* CKOs, affecting one-third of the diplotene cell population. Thus, HUS1 is required for some aspect of chromosome integrity that affects the SC, the failure of which may contribute to the high level of metaphase germ cell loss. This SC rupture phenotype (Figure 5C; Table 2) is particularly striking and may be a key driver of the fertility defect observed following *Hus1* loss, as no significant change was observed in the number of MLH1 foci in *Hus1* mutant pachytene spermatocytes or in the number of bivalents seen at diakinesis (Figure S6). The molecular cause of the SC rupture defect remains unresolved, although it could be related to incomplete repair of meiotic DSBs.

It is still unclear why *Hus1* CKO cells have a small number of persistent DSBs, and why many of these are associated with death at metaphase I. Perhaps the 4–8 remaining unrepaired breaks are not sufficient to trigger the pachytene checkpoint (or cannot, due to a requirement for HUS1), but lead to a defect that is recognized at metaphase by the spindle checkpoint. It is possible that the persistent breaks are not simple D-loop intermediates containing RAD51 and γH2AX, but are complex multi-chromatid intermediates, such as those seen in *Blm/Sgs1* helicase mutants [71], [72]. The symmetrical RAD51 foci seen on either side of the chromosome cores in 9% of diplotene nuclei from *Hus1* CKOs (see Figure 4, asterisks) would support this, pointing to RAD51/DMC1 presence on more than one homolog during homolog separation. Clearly, the extent of the DNA repair defect in *Hus1* CKO mice is less severe than that observed in other meiotic mutants. For instance, *Trip13* mutant spermatocytes have been reported to exhibit between 99 and 138 RAD51 foci during pachytene compared to 11–18 foci in controls [54], [73], whereas we observed an average of 25 RAD51 foci in early to mid-pachytene *Hus1* CKOs (compared to 22 foci in controls) and 9 RAD51 foci in late pachytene (compared to 2 foci/nucleus in controls). These results are consistent with the idea that HUS1 has a relatively late and restricted role in DSB repair, perhaps related to the completion of a subset of late recombination events.

The persistent γH2AX and RAD51 foci on *Hus1-*deficient meiotic chromosomes as well as the colocalization of RAD9 with RAD51 on normal chromosomes suggest that the 9-1-1 complex is critical for the efficient repair of a subset of meiotic DSBs. Several lines of evidence in somatic cells also indicate that 9-1-1 functions directly in homologous recombination (HR) repair of DSBs. In human cells, 9-1-1 is reported to physically interact with RAD51, and *Rad9* knockdown results in reduced HR [7]. In a separate system using human cells with conditional *Rad9* repression, RAD9 enhances survival and DNA repair in response to ionizing radiation [74], and similarly, mouse *Rad9* ^−/−^ ES cells are sensitive to γ-irradiation [43]. Reducing *Hus1* expression in mouse cells via siRNA also decreases the efficiency of HR repair [75]. In contrast to the situation in yeast where 9-1-1 is proposed to be important for the loading or assembly of RAD51 complexes onto meiotic chromosomes [37], we propose that mammalian 9-1-1 may be important for later steps of meiotic recombination, since we did not observe delayed loading of RAD51, and RAD51 foci persisted in the absence of *Hus1*. Among the known 9-1-1 binding partners are DNA ligase I [13] and DNA Polymerase β [9], [10], the latter of which is known to play critical roles during mammalian meiosis [76], [77], raising the possibility that 9-1-1 may recruit Pol β and DNA ligase to recombination intermediates to complete repair. Alternatively, interactions between the 9-1-1 complex and translesion synthesis polymerases [8], which have been implicated in HR in somatic cells [78]–[80], could promote the extension of the 3′ ends subsequent to RAD51-mediated strand exchange. The requirement for 9-1-1 could be accentuated due to unique features of genome maintenance in meiotic cells. For instance, non-homologous end joining, a major mechanism for DSB repair in somatic cells, is suppressed during meiosis [29], [81]. In addition, the unique structure of SPO11-induced meiotic DSBs may create a greater demand for 9-1-1 complex-mediated repair functions than a typical mitotic DSB. The repair of most meiotic DSBs occurred normally in the absence of HUS1, suggesting that the 9-1-1 complex may be necessary to deal with only a subset of breaks, perhaps ones that prove difficult to repair because of the sequence context, chromatin structure, or other factors.

The production of haploid gametes during meiosis clearly raises challenges for genome maintenance, many of which are distinct from those in somatic cells. Based on the findings reported here, we propose that mammalian 9-1-1 components have acquired specialized roles during meiosis, with the canonical RAD9-RAD1-HUS1 complex functioning at DSBs and an alternative RAD1-containing complex functioning at sites of asynapsis. The mouse models described here represent powerful systems to elucidate how the mammalian 9-1-1 complex promotes meiotic chromosome integrity, in some cases distinct from the well-established roles of 9-1-1 in TOPBP1- and ATR-dependent checkpoint signaling, and highlight the intriguing possibility of alternative checkpoint clamps functioning in various capacities in the mammalian germline.

## Materials and Methods

### Ethics statement

All animals used in this study were handled in accordance with federal and institutional guidelines, under a protocol approved by the Cornell University Institutional Animal Care and Use Committee (IACUC).

### Mouse strains and husbandry

Mice harboring two conditional *Hus1* alleles (*Hus1^flox/flox^*) were crossed to *Cre*-positive mice harboring one null *Hus1* allele (*Stra8-Cre+ Hus1^+/^* ^Δ*1*^ or *Spo11-Cre+ Hus1^+/^* ^Δ*1*^) to generate experimental germ cell-specific *Hus1* conditional knockout mice (*Cre*+ *Hus1^flox/^* ^Δ*1*^) as well as littermate control animals (*Cre+ Hus1^+/flox^* and *Cre- Hus1^flox/^* ^Δ*1*^), as shown in Figure S1A. Conditional and null *Hus1* alleles were described previously [46]. *Stra8-Cre* transgenic FVB mice were kindly provided by Bob Braun (The Jackson Laboratory; [47]). *Spo11-Cre* mice were generated as described in Figure S2 and in Text S1. *Msh4^−/−^* mice were kindly provided by Winfried Edelmann (Albert Einstein College of Medicine; [66]), and *Dmc1^−/−^* [65] and *Spo11^−/−^* [64] mutant mice were generously provided by John Schimenti (Cornell University). *Slx4^mut/mut^* mice were derived from the previously reported *Btbd12^tm1a(EUCOMM)Wtsi^* strain and carried an intact β-geo cassette and germline *Slx4* exon 3 deletion [55]. For fertility testing, 8- to 12-week old *Stra8-Cre Hus1* CKO and control males were singly housed with wild-type 129 or FVB females. Copulatory plugs were monitored daily, and plugged females were removed to separate cages and monitored for pregnancy. Viable pups were counted on the first day of life.

### Western blotting

Flash frozen testes from 17-day old, 20-day old, or adult animals (12–14 weeks, unless labeled otherwise) were homogenized in RIPA buffer supplemented with protease inhibitors and sodium orthovanadate using a Tissuelyzer, sonicated at 24–27W twice for 2 minutes each, then cleared by centrifugation. Antibodies included rabbit polyclonal anti-HUS1 HM199 (rabbit polyclonal antiserum generated against purified recombinant GST-tagged full-length mouse HUS1 protein), anti-phosphoCHK1 (Ser345; Cell Signaling #2341), anti-CHK1 (Santa Cruz), anti-CHK2 (clone 7, Millipore #05-649), anti-RAD9 HM456 (rabbit polyclonal antiserum generated against purified recombinant HIS-tagged full-length mouse RAD9 protein), affinity purified rabbit anti-RAD1 [41], and anti-β-actin (Sigma).

### Histology and immunohistochemistry

Testes were fixed overnight at 4°C in Bouin's fixative (for H&E and GCNA1 staining) or at room temperature in 10% neutral-buffered formalin (for TUNEL staining), embedded in wax, and sectioned at 5 µm. Immunohistochemical staining for germ cell nuclear antigen [GCNA1; 82] was performed using anti-GCNA1 antibody provided by George Enders. TUNEL assays were performed using the Apoptag kit (Millipore) as per the manufacturer's instructions. TUNEL data were quantified by counting the number of TUNEL-positive cells per tubule in at least 50 tubules from the testes of at least 3 different mice of each genotype, and differences between controls and *Hus1* CKOs were analyzed statistically via Student's *t*-test.

### Epididymal sperm counts

Both caudal epididymides from each 12-week old mouse were minced with fine forceps in a petri dish with 37°C PBS, incubated, and fixed in 10% neutral-buffered formalin (1∶25 dilution). Sperm counts shown in Figure 1 are the mean of 6 to 9 mice per group ± SEM, analyzed statistically using a Student's *t*-test.

### Meiotic chromosome spreading and immunofluorescence staining

Surface-spread nuclei were prepared from 12-week old male mice as described previously [58], with the exception of *Dmc1^−/−^* and control littermate samples which were prepared similarly from flash-frozen testes. Briefly, tubules were incubated in hypotonic extraction buffer on ice for 1 hour, minced in 100 mM sucrose, and spread on slides dipped in 1% PFA with 0.15% Triton X-100. Slides were incubated in a humid chamber for 2.5 hours, dried, and washed in PBS and water containing Photoflo (Kodak). Immunofluorescence staining was performed following blocking in 10% goat or donkey serum and 3% BSA, with primary antibodies incubated overnight at room temperature and secondary antibodies incubated at 37°C for one hour in the dark. Slides were mounted with coverslips using homemade anti-fade mounting medium (2.3% DABCO, 20 mM Tris pH 8.0, 8 µg DAPI in 90% glycerol).

Primary antibodies used for immunofluorescence staining included those recognizing: γH2AX (1∶5000; Upstate/Millipore), SYCP1 (1∶500), SYCP3 (1∶5000; [83]), RAD9 HM456 (1∶600; see above), RAD1 (1∶17; [41]), TOPBP1 (1∶500; [84]), RAD51 (1∶500; Oncogene Research Products/EMD Biosciences), RNA Polymerase II (1∶500; Millipore), and MLH1 (1∶50; BD Pharmingen). For co-staining of RAD9 and RAD1, we additionally used affinity purified sheep anti-RAD1 (sheep polyclonal antiserum generated against purified recombinant 6X HIS full-length human RAD1) generated in the Freire laboratory, and for co-staining of RAD9 with RAD51, we used mouse monoclonal anti-RAD51 (Abcam). For MLH1/SYCP3/RAD51 co-staining, anti-SYCP3 antibody was used at 1∶50,000 as described Lipkin *et al.* [85]. Human CREST serum was used to detect centromeres in some experiments as previously described [86]. Secondary antibodies were used at 1∶1000 dilution and included goat anti-mouse Alexafluor 488, goat anti-rabbit Alexafluor 555, donkey anti-sheep Alexafluor 488, and donkey anti-rabbit Alexafluor 555 (Invitrogen). Microscopy and imaging was performed as described previously [56].

For quantification of phenotypes shown in Table 2, a “grossly normal” nucleus was defined as one with normal synapsis and γH2AX staining confined to the sex body, and an “abnormal sex body” was defined as one with extended γH2AX signal, inclusion of whole or partial autosomes, and/or the presence of an apparent X chromosome-autosome end-to-end fusion. P-values for differences in γH2AX staining (Table 2), sex body abnormalities (Table 2), and synapsis defects (text) were calculated using a *z*-test of sample proportions, comparing *Hus1* CKOs to control animals. Diakinesis chromosome spreads (Figure S6D) were generated as described previously [56] from two independent 4- to 5-week old mice per genotype.

## Supporting Information

Figure S1 *Hus1* alleles and *Cre-*expressing mice used in this study. A. Breeding scheme for generating *Hus1* conditional knockout animals. CRE-mediated excision acting upon the *Hus1^flox^* allele resulted in the null *Hus1* ^Δ*2,3*^ allele. B. *Cre*-expressing mouse lines used in this study. *Stra8-Cre* is expressed beginning at postnatal day 3 in undifferentiated spermatogonia through leptotene spermatocytes [47]. *Spo11-Cre* is expressed beginning at postnatal day 10 in primary spermatocytes (Figure S2; Text S1). C,D. *Hus1* deletion was detected in total testis DNA from 4-week old *Stra8-Cre* (C) and *Spo11-Cre* (D) *Hus1* CKO mice, but little deletion was detected in spleen.
(TIF)

Figure S2 Generation and characterization of *Spo11-Cre*-expressing mice. A. Construct used to generate *Spo11-eGFP-Cre* animals. B. Fluorescence (tRFP, GFP, DAPI) and immunohistochemical staining (anti-GFP) of testis sections from animals with or without an *mTmG* reporter and/or *Spo11-eGfp-Cre* transgene. GFP-tagged *Spo11-Cre* was detectable in spermatocytes. CRE-mediated recombination in *mTmG*+ spermatocytes resulted excision of the RFP cassette and expression of the GFP reporter in late-stage germ cells. Anti-GFP immunohistochemistry also detected GFP in spermatocytes (from expression of GFP-tagged *Spo11-Cre*) and later-stage germ cells (from the CRE-recombined *mTmG* reporter). C. PCR-based detection of RFP (from undeleted *mTmG*+ animals), GFP (in *Spo11-Cre*+ animals as well as in recombined *mTmG* animals), and Actin from cDNA of animals negative or positive for *mTmG* and/or *Spo11-Cre*. D. Southern blot of DNA extracted from testis of 10-day and 12-week old *Spo11-Cre*+ animals, either *Hus1^+/flox^* or *Hus1^flox/^* ^Δ*1*^. CRE-mediated deletion was detectable in the testis as early as postnatal day 10, and nearly complete deletion was observed in adult animals. The small amount of *Hus1^flox^* remaining may derive from non-germ cells and spermatogonia, which do not express *Spo11-Cre*.
(TIF)

Figure S3 *Hus1* deletion results in alterations in HUS1, RAD9, and RAD1 protein levels as well as altered levels of CHK1, pCHK1, and pCHK2. Testis lysates from control and *Hus1* CKO mice (*Stra8-Cre* or *Spo11-Cre* as indicated) at the indicated ages were subjected to Western blotting. A. Total protein levels of both the RAD9 and RAD1 subunits were significantly reduced in adult mice lacking *Hus1*. B. HUS1 and RAD1 protein levels were significantly altered in testes from *Stra8-Cre Hus1* CKO mice at as early as 17 days of age. C. HUS1 protein levels were more subtly reduced in *Spo11-Cre Hus1* CKO testes, despite efficient genomic *Hus1* deletion. Consistent with a more subtle reduction in HUS1 protein levels in *Spo11-Cre* versus *Stra8-Cre Hus1* CKO mice, immunofluorescence staining of meiotic chromosome spreads revealed that RAD9 foci were detectable in 49% of early- to mid-pachytene *Spo11-Cre Hus1* CKOs compared to 95% of controls (N = 42 and 72, respectively), whereas 99% of pachytene cells in *Stra8-Cre Hus1* CKOs lacked detectable RAD9 foci.
(TIF)

Figure S4 Germ cell loss is apparent at 17 days in *Stra8-Cre Hus1* CKO animals. A. TUNEL staining of 17-day testes from control (*Cre-*negative *Hus1^+/flox^*) and *Stra8-Cre Hus1* CKO males, indicating increased apoptosis of spermatogonia and spermatocytes in the absence of *Hus1*. B. Quantification of TUNEL staining from at least 3 animals per genotype, displayed as the mean ± SEM. The asterisk indicates a statistically significant increase in apoptosis as determined by Student's *t*-test (p<0.05).
(TIF)

Figure S5 *Hus1* inactivation using *Spo11-Cre* results in germ cell loss. A. H&E-stained sections from 12-week old control (*Cre+ Hus1^+/flox^*) and *Spo11-Cre Hus1* CKO males. Arrows indicate multinucleate spermatid giant cells. B. Images of TUNEL-stained 12-week old males, indicating increased apoptosis in *Spo11-Cre Hus1* CKOs. C. Quantification of TUNEL staining shown in B, displayed as the mean ± SEM. The asterisk indicates a statistically significant increase in apoptosis as determined by Student's *t*-test (p<0.05).
(TIF)

Figure S6 MLH1-dependent crossovers and diakinesis chromosomes appear normal in the absence of *Hus1*. A. Normal MLH1 localization in control and *Hus1* CKO pachytene spermatocytes. B. Quantification of MLH1 foci from 3 individual mice per genotype; N = 93 and N = 97 for control and *Hus1* CKO, respectively. C. Coimmunofluorescence staining of RAD51, MLH1, and SYCP3 in *Stra8-Cre Hus1* CKO nuclei with persistent RAD51 foci. Arrow indicates rare colocalization of a persistent RAD51/DMC1 focus with MLH1. D. Representative images of Giemsa-stained diakinesis chromosome spreads from control and *Hus1* CKO testes. E. Quantification of bivalents and aberrant nuclei (those containing univalents or chromosomal fragments) from 47 control and 32 *Hus1* CKO diakinesis chromosome spreads, derived from two mice per genotype.
(TIF)

Figure S7 RAD9 localization is dependent upon meiotic DSBs. Meiotic chromosome spreads from control, *Spo11^−/−^*, or *Dmc1* ^−/−^ mice were stained for RAD9 and SYCP3. RAD9 localization is abundant along normal zygotene chromosomes (A), is reduced in the absence of meiotic DSBs in *Spo11^−/−^* mutants (B), and is increased in the presence of increased unrepaired DSBs in *Dmc1* ^−/−^ mutants (C).
(TIF)

Text S1 Additional methodological details and related references.
(DOCX)
