# Supplementary figures and images for: Conditional Inactivation of the DNA Damage Response Gene Hus1 in Mouse Testis Reveals Separable Roles for Components of the RAD9-RAD1-HUS1 Complex in Meiotic Chromosome Maintenance
Source: PLoS Genet. 2013 Feb 28;9(2):e1003320. doi: 10.1371/journal.pgen.1003320 (PMC3585019; doi:10.1371/journal.pgen.1003320)

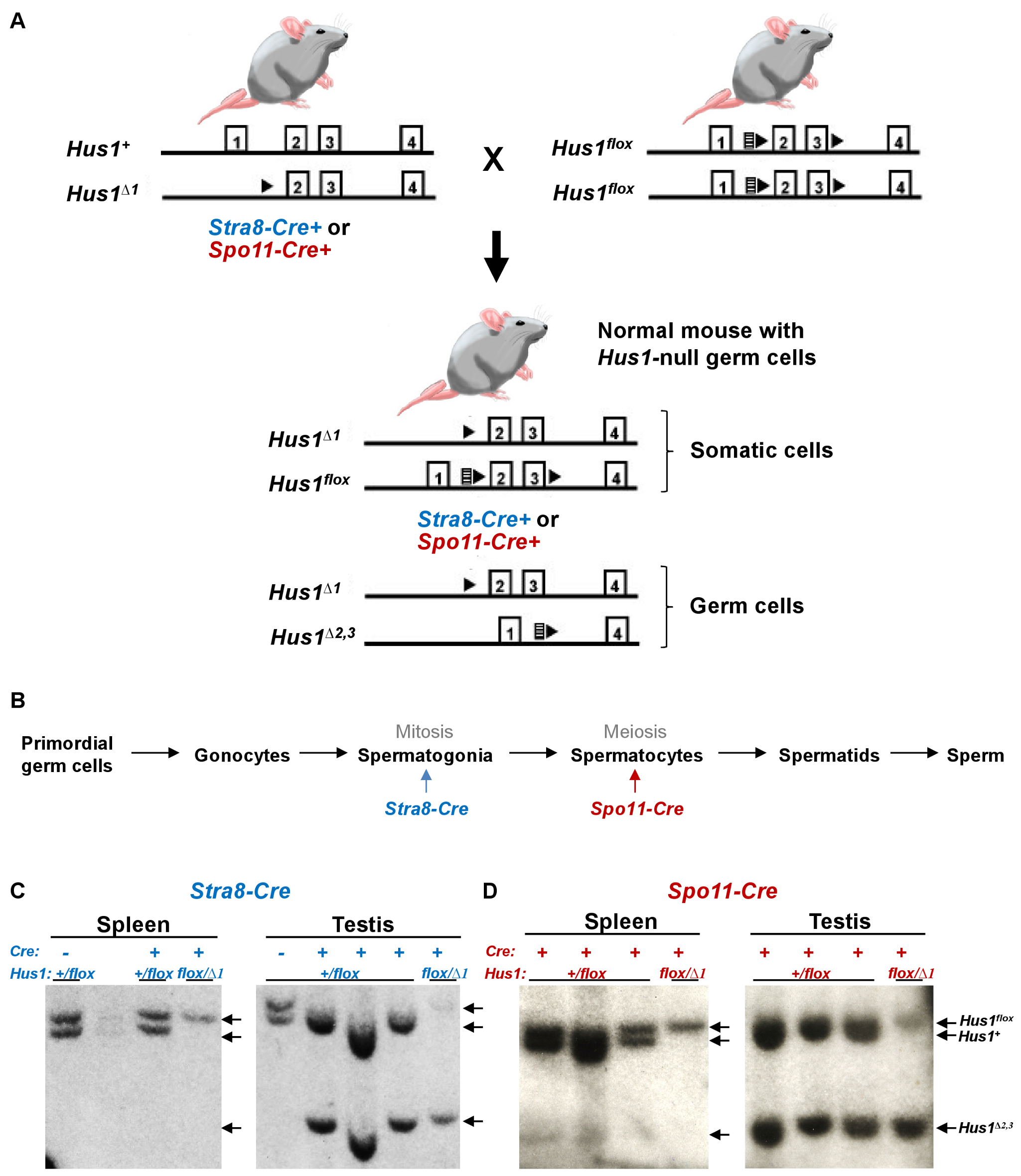

Supplement: Figure S1 — Hus1 alleles and Cre-expressing mice used in this study. A. Breeding scheme for generating Hus1 conditional knockout animals. CRE-mediated excision acting upon the Hus1flox allele resulted in the null Hus1 Δ2,3 allele. B. Cre-expressing mouse lines used in this study. Stra8-Cre is expressed beginning at postnatal day 3 in undifferentiated spermatogonia through leptotene spermatocytes [47]. Spo11-Cre is expressed beginning at postnatal day 10 in primary spermatocytes (Figure S2; Text S1). C,D. Hus1 deletion was detected in total testis DNA from 4-week old Stra8-Cre (C) and Spo11-Cre (D) Hus1 CKO mice, but little deletion was detected in spleen. (TIF) [file pgen.1003320.s001.tif]

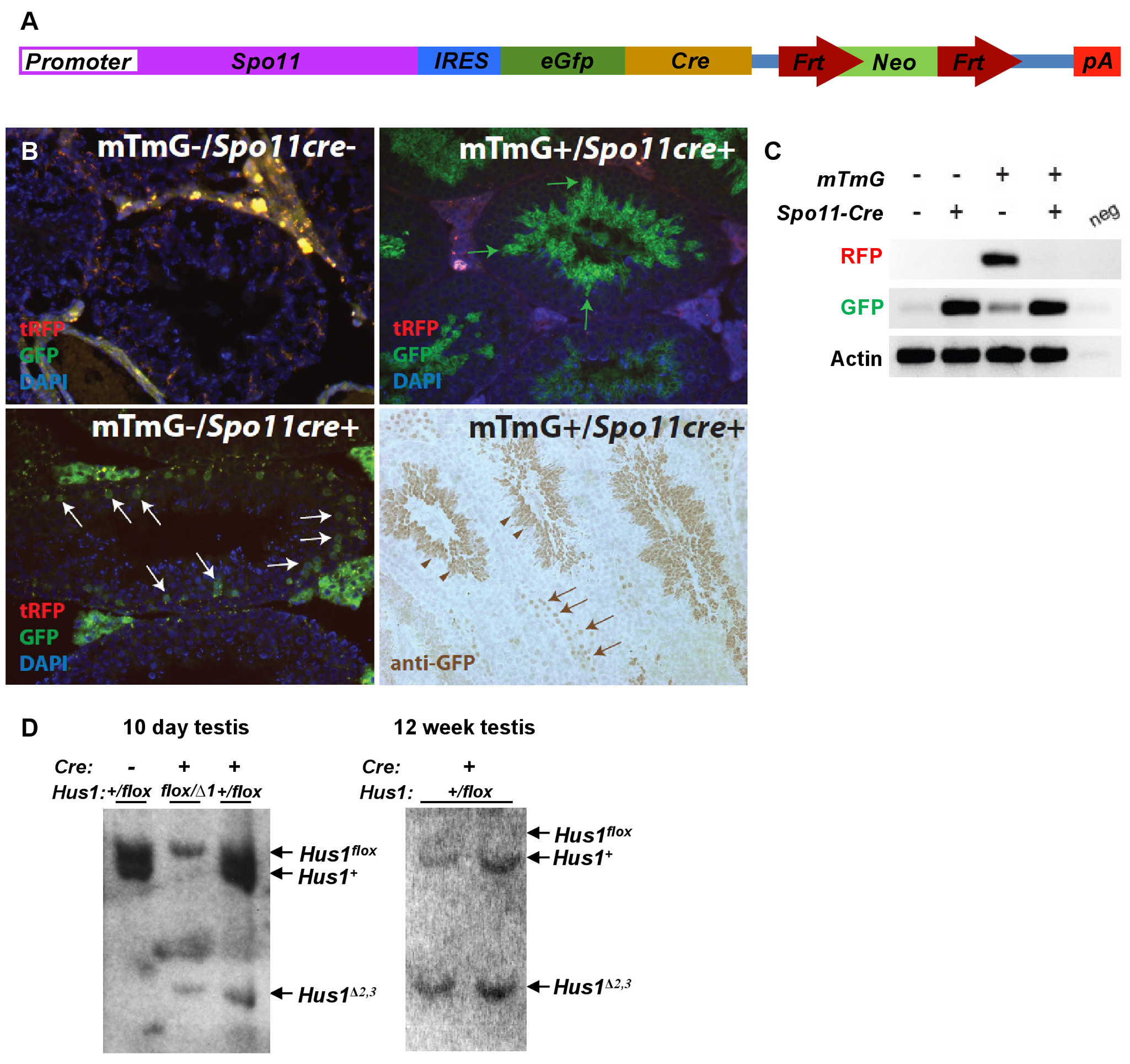

Supplement: Figure S2 — Generation and characterization of Spo11-Cre-expressing mice. A. Construct used to generate Spo11-eGFP-Cre animals. B. Fluorescence (tRFP, GFP, DAPI) and immunohistochemical staining (anti-GFP) of testis sections from animals with or without an mTmG reporter and/or Spo11-eGfp-Cre transgene. GFP-tagged Spo11-Cre was detectable in spermatocytes. CRE-mediated recombination in mTmG+ spermatocytes resulted excision of the RFP cassette and expression of the GFP reporter in late-stage germ cells. Anti-GFP immunohistochemistry also detected GFP in spermatocytes (from expression of GFP-tagged Spo11-Cre) and later-stage germ cells (from the CRE-recombined mTmG reporter). C. PCR-based detection of RFP (from undeleted mTmG+ animals), GFP (in Spo11-Cre+ animals as well as in recombined mTmG animals), and Actin from cDNA of animals negative or positive for mTmG and/or Spo11-Cre. D. Southern blot of DNA extracted from testis of 10-day and 12-week old Spo11-Cre+ animals, either Hus1+/flox or Hus1flox/ Δ1. CRE-mediated deletion was detectable in the testis as early as postnatal day 10, and nearly complete deletion was observed in adult animals. The small amount of Hus1flox remaining may derive from non-germ cells and spermatogonia, which do not express Spo11-Cre. (TIF) [file pgen.1003320.s002.tif]

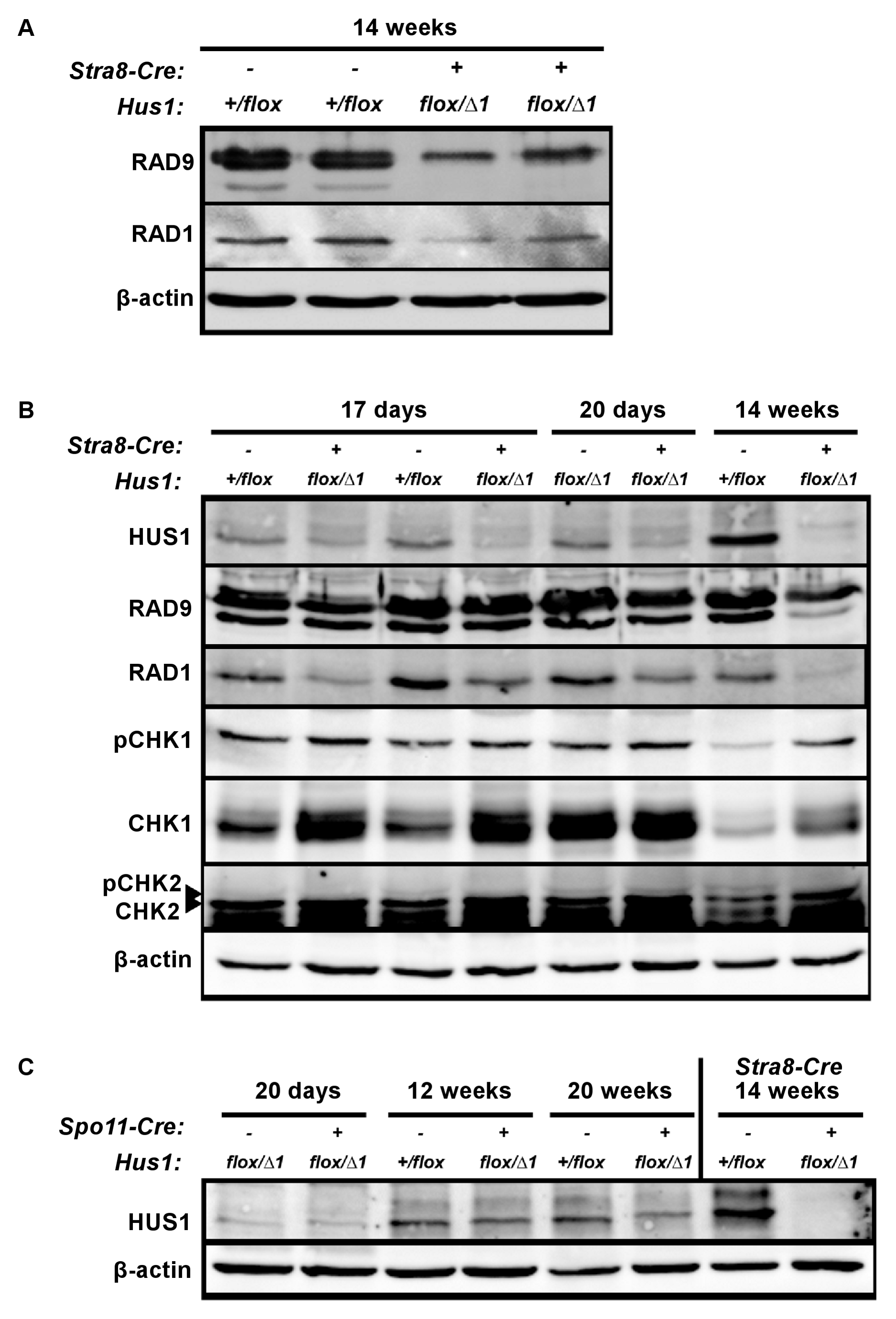

Supplement: Figure S3 — Hus1 deletion results in alterations in HUS1, RAD9, and RAD1 protein levels as well as altered levels of CHK1, pCHK1, and pCHK2. Testis lysates from control and Hus1 CKO mice (Stra8-Cre or Spo11-Cre as indicated) at the indicated ages were subjected to Western blotting. A. Total protein levels of both the RAD9 and RAD1 subunits were significantly reduced in adult mice lacking Hus1. B. HUS1 and RAD1 protein levels were significantly altered in testes from Stra8-Cre Hus1 CKO mice at as early as 17 days of age. C. HUS1 protein levels were more subtly reduced in Spo11-Cre Hus1 CKO testes, despite efficient genomic Hus1 deletion. Consistent with a more subtle reduction in HUS1 protein levels in Spo11-Cre versus Stra8-Cre Hus1 CKO mice, immunofluorescence staining of meiotic chromosome spreads revealed that RAD9 foci were detectable in 49% of early- to mid-pachytene Spo11-Cre Hus1 CKOs compared to 95% of controls (N = 42 and 72, respectively), whereas 99% of pachytene cells in Stra8-Cre Hus1 CKOs lacked detectable RAD9 foci. (TIF) [file pgen.1003320.s003.tif]

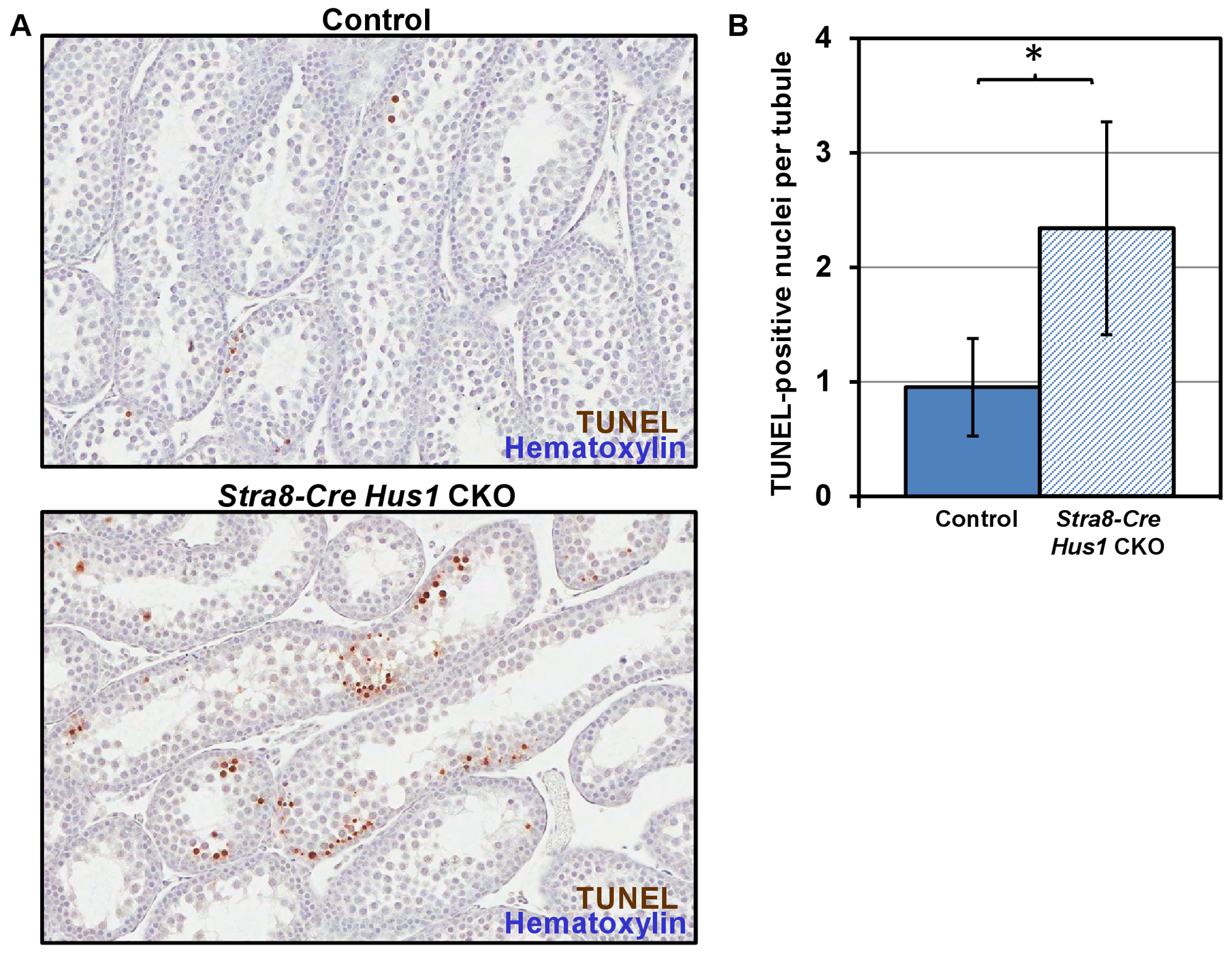

Supplement: Figure S4 — Germ cell loss is apparent at 17 days in Stra8-Cre Hus1 CKO animals. A. TUNEL staining of 17-day testes from control (Cre-negative Hus1+/flox) and Stra8-Cre Hus1 CKO males, indicating increased apoptosis of spermatogonia and spermatocytes in the absence of Hus1. B. Quantification of TUNEL staining from at least 3 animals per genotype, displayed as the mean ± SEM. The asterisk indicates a statistically significant increase in apoptosis as determined by Student's t-test (p<0.05). (TIF) [file pgen.1003320.s004.tif]

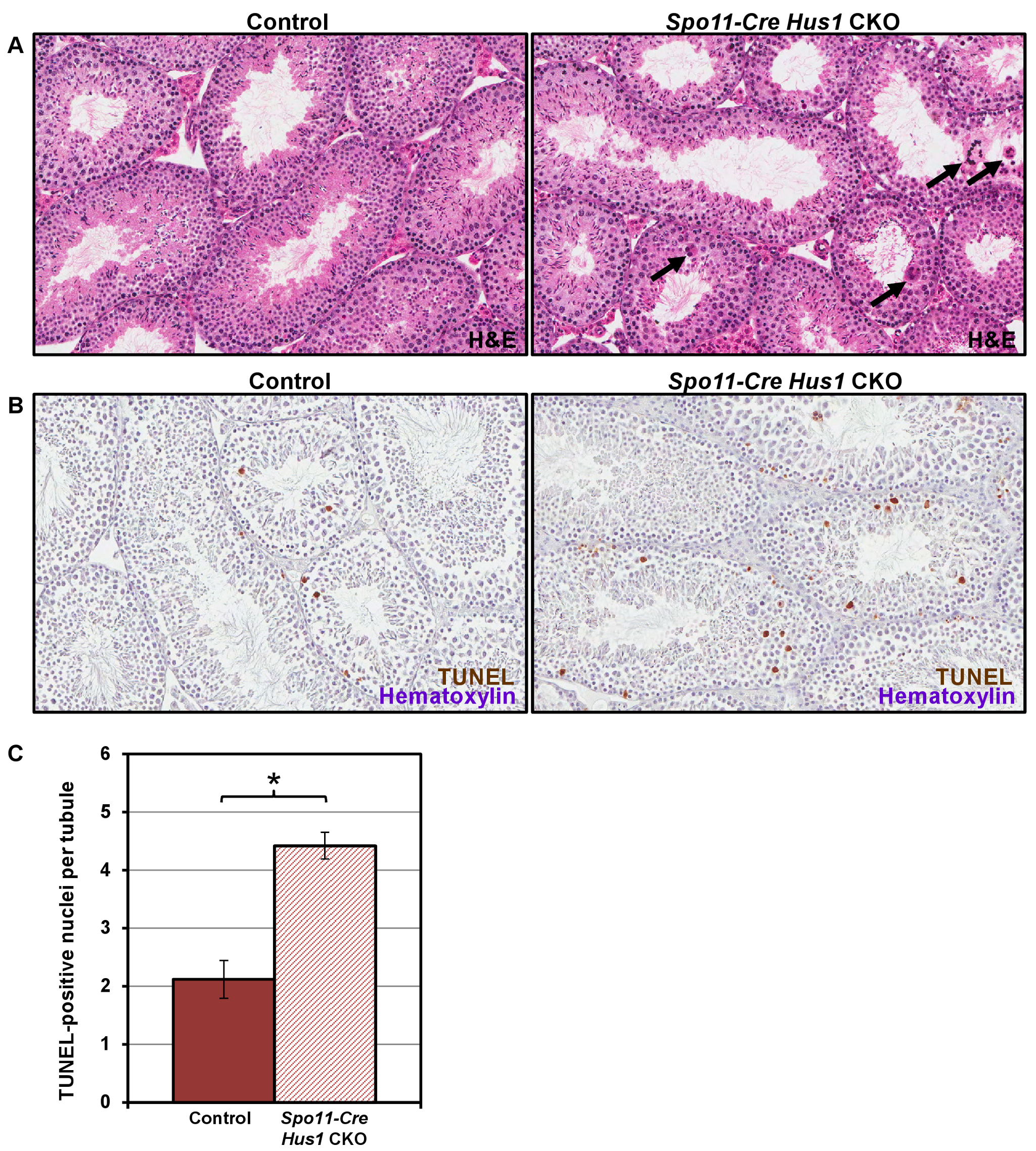

Supplement: Figure S5 — Hus1 inactivation using Spo11-Cre results in germ cell loss. A. H&E-stained sections from 12-week old control (Cre+ Hus1+/flox) and Spo11-Cre Hus1 CKO males. Arrows indicate multinucleate spermatid giant cells. B. Images of TUNEL-stained 12-week old males, indicating increased apoptosis in Spo11-Cre Hus1 CKOs. C. Quantification of TUNEL staining shown in B, displayed as the mean ± SEM. The asterisk indicates a statistically significant increase in apoptosis as determined by Student's t-test (p<0.05). (TIF) [file pgen.1003320.s005.tif]

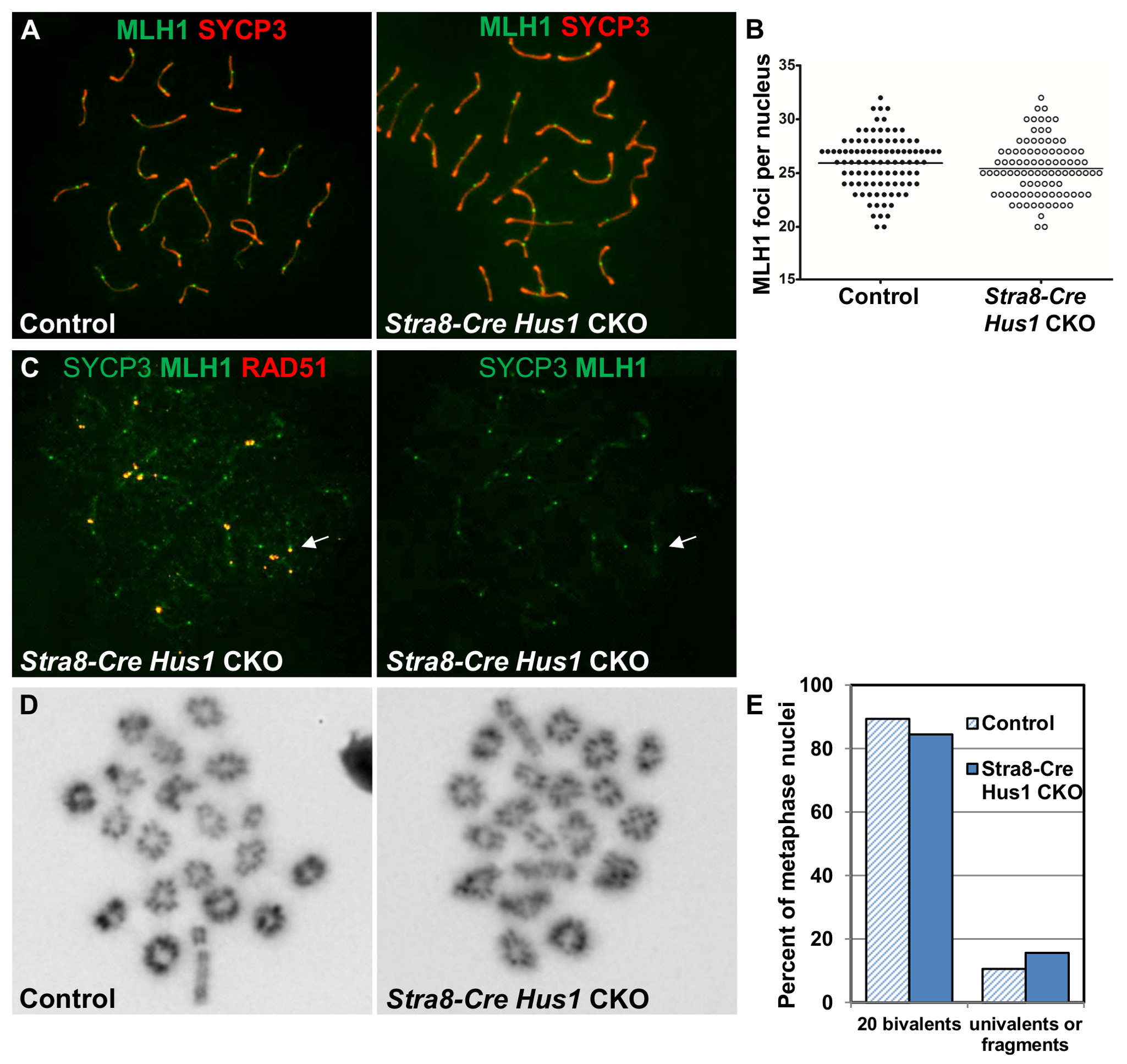

Supplement: Figure S6 — MLH1-dependent crossovers and diakinesis chromosomes appear normal in the absence of Hus1. A. Normal MLH1 localization in control and Hus1 CKO pachytene spermatocytes. B. Quantification of MLH1 foci from 3 individual mice per genotype; N = 93 and N = 97 for control and Hus1 CKO, respectively. C. Coimmunofluorescence staining of RAD51, MLH1, and SYCP3 in Stra8-Cre Hus1 CKO nuclei with persistent RAD51 foci. Arrow indicates rare colocalization of a persistent RAD51/DMC1 focus with MLH1. D. Representative images of Giemsa-stained diakinesis chromosome spreads from control and Hus1 CKO testes. E. Quantification of bivalents and aberrant nuclei (those containing univalents or chromosomal fragments) from 47 control and 32 Hus1 CKO diakinesis chromosome spreads, derived from two mice per genotype. (TIF) [file pgen.1003320.s006.tif]

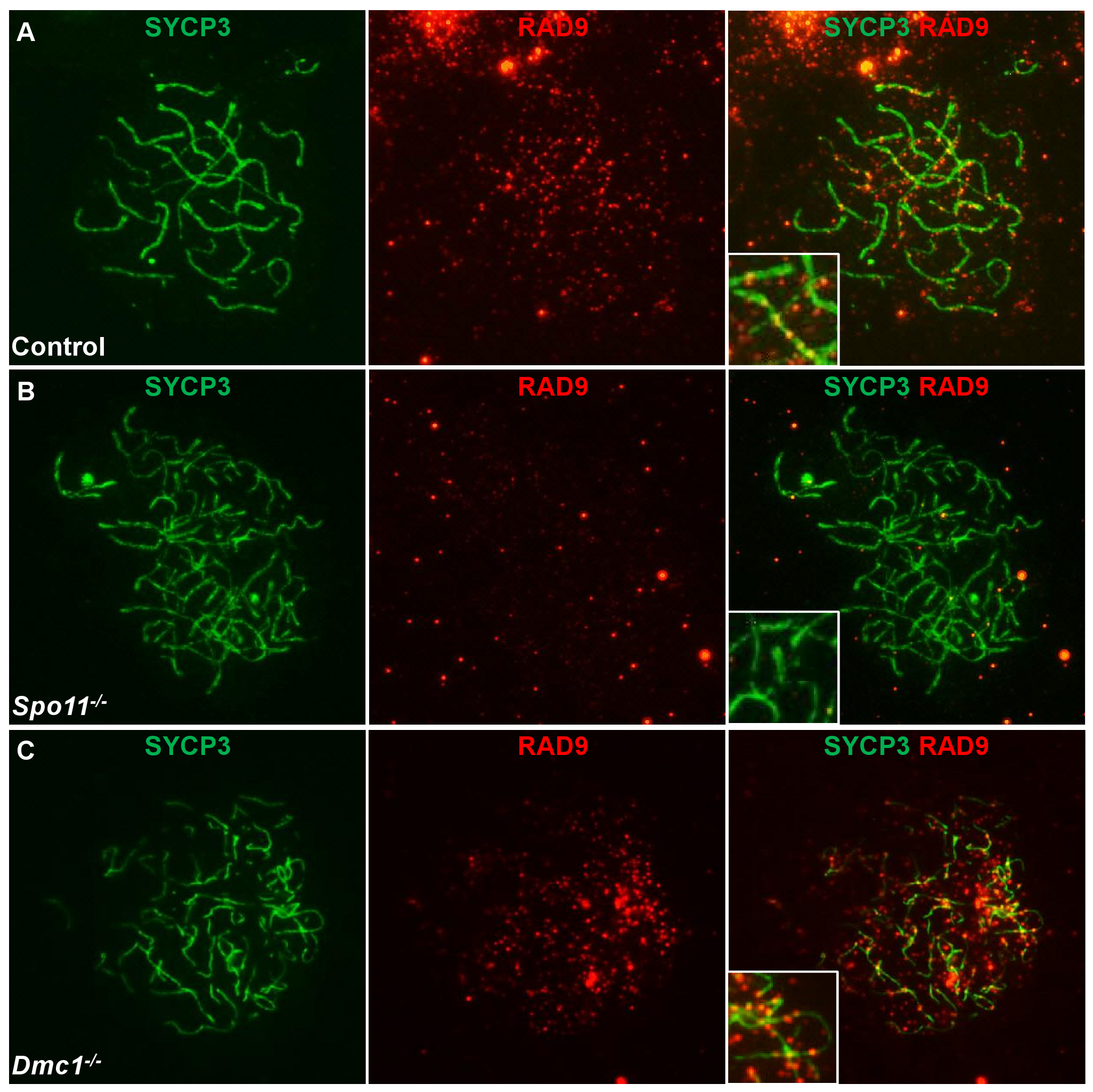

Supplement: Figure S7 — RAD9 localization is dependent upon meiotic DSBs. Meiotic chromosome spreads from control, Spo11−/−, or Dmc1 −/− mice were stained for RAD9 and SYCP3. RAD9 localization is abundant along normal zygotene chromosomes (A), is reduced in the absence of meiotic DSBs in Spo11−/− mutants (B), and is increased in the presence of increased unrepaired DSBs in Dmc1 −/− mutants (C). (TIF) [file pgen.1003320.s007.tif]
