# Supplementary material for: Conditional Inactivation of the DNA Damage Response Gene Hus1 in Mouse Testis Reveals Separable Roles for Components of the RAD9-RAD1-HUS1 Complex in Meiotic Chromosome Maintenance
Source: PLoS Genet. 2013 Feb 28;9(2):e1003320. doi: 10.1371/journal.pgen.1003320 (PMC3585019; doi:10.1371/journal.pgen.1003320)
Supplement: Text S1 — Additional methodological details and related references. (DOCX) [file pgen.1003320.s008.docx]

**Supporting Information Text S1**

**Generation of *Spo11-Cre* transgenic mice.** The *Spo11-Cre* construct was generated by inserting an *IRES-eGfp-Cre* cassette (kindly provided by Dr. Michael Kotlikoff, Cornell University) and an *Frt*-flanked *Neo* cassette into a BAC (RP23-20N4) containing the entire mouse *Spo11* genomic locus. A fragment containing the *Spo11* gene, including approximately 5kb of upstream sequences, as well as the inserted *IRES-eGfp-Cre* and *Frt*-flanked *Neo* cassettes was excised from the modified BAC and electroporated into ES cells, followed by G418 selection. Genomic insertion was verified by PCR analysis. The transgene-containing ES cells were injected into blastocysts using standard methods, and the *Frt*-flanked *Neo* marker removed by crossing the resultant mice with *Actb-FLPe* mice to produce *Spo11-eGFP-Cre* (referred to here as *Spo11-Cre*) mice. *Spo11-Cre* mice were initially backcrossed onto a C57BL/6 background, but backcrossed to 129 in the course of the experiments described here.

**Characterization of *Spo11-Cre* function.**  In order to test the specificity of CRE recombinase activity in *Spo11-Cre* mice, we crossed them to *mTmG* reporter mice in which CRE-mediated excision results in loss of expression of a membrane-associated Tomato red reporter cassette (mT) and activation of a membrane-associated GFP reporter (mG) [[1](#_ENREF_1)]. Bright GFP signal (enhanced above the level of green fluorescence produced by *Spo11-Cre-eGFP* alone) was detectable in the germ cells of *Spo11-Cre+ mTmG* mice following CRE-mediated excision (Figure S2B). These results were confirmed by PCR detection of Tomato/tRFP expression loss and GFP gain in cDNA generated from *Spo11-Cre+ mTmG* and control mice (Figure S2C), and further by Southern blotting for the excised *Hus1* allele in testes from 10-day (Figure S2D), 4-week (Figure S1D), and 12-week old (Figure S2D) mice. For Southern blotting, DNA was prepared from frozen tissues and blotted as described previously [[2](#_ENREF_2)].

**Supplemental References**

1. Muzumdar MD, Tasic B, Miyamichi K, Li L, Luo L (2007) A global double-fluorescent Cre reporter mouse. Genesis 45: 593-605.

2. Yazinski SA, Westcott PMK, Ong K, Pinkas J, Peters RM, et al. (2009) Dual inactivation of Hus1 and p53 in the mouse mammary gland results in accumulation of damaged cells and impaired tissue regeneration. Proc Natl Acad Sci USA 106: 21282-21287.
